# Single-cell and spatial multi-omics reveal estrogen-mediated vaginal wall microenvironment remodeling and a perivascular reparative niche in postmenopausal pelvic organ prolapse

**DOI:** 10.3389/fimmu.2026.1794699

**Published:** 2026-07-03

**Authors:** Lin Wang, Lingyun Wei, Mengyu Geng, Shuyu Wang, Wenzhen Wang, Nan Jia, Xiaochun Liu

**Affiliations:** 1 Third Hospital of Shanxi Medical University, Shanxi Bethune Hospital, Shanxi Academy of Medical Sciences, Tongji Shanxi Hospital, Taiyuan, China; 2 Shanxi Bethune Hospital, Shanxi Academy of Medical Sciences, Third Hospital of Shanxi Medical University, Tongji Shanxi Hospital, Taiyuan, China

**Keywords:** fibroblast reprogramming, pelvic organ prolapse, perivascular niche, single-cell RNA sequencing, spatial transcriptomics

## Abstract

**Introduction:**

Local estrogen is widely used to improve vaginal mucosal status and relieve menopausal urogenital symptoms in patients with pelvic organ prolapse (POP), yet clinical outcomes are inconsistent. This creates an urgent need to clarify the tissue-specific mechanisms of estrogen action.

**Methods:**

In this study, we combined single-cell RNA sequencing and high-resolution Visium HD spatial transcriptomics to profile postmenopausal vaginal wall tissues at near-cellular resolution. Computational pharmacology analysis was also performed to predict distinct responses of this tissue niche to different estrogen subtypes.

**Results:**

We found estrogen drives selective expansion and spatial redistribution of HAS1+ fibroblasts, which aggregate with pericytes to form a structured perivascular niche. Such spatial co-localization strengthens fibroblast-pericyte crosstalk and activates multiple pro-repair signaling cascades.

**Discussion:**

Our results demonstrate that estrogen functions by forming spatially organized multicellular reparative niches rather than non-selective tissue activation. This work provides a theoretical basis for developing targeted POP therapies, while further experimental verification is still needed

## 1 Introduction

Pelvic organ prolapse (POP) is a common pelvic floor disorder that severely impacts the quality of life for middle-aged and elderly women and represents a significant public health burden. Epidemiological studies reveal that nearly half of all women exhibit some degree of anatomical POP (1). For patients with severe symptoms, surgery is the primary treatment; however, the choice of surgical procedure is complex and carries a risk of recurrence (2), with approximately 11–19% of patients requiring surgical intervention (3). Notably, about 30% of surgical cases may require reoperation due to recurrence (1, 4), reflecting the chronic and refractory nature of the disease. Clinically, POP often presents with a range of symptoms such as pelvic pressure, urinary dysfunction, and decreased sexual quality of life, which not only harm patients’ physical and mental health but also impose a substantial socioeconomic burden—for instance, the annual direct medical cost for POP in the United States exceeds $1.5 billion (5). With the accelerating global aging population, the total number of patients is projected to increase by 46% to approximately 156 million by 2050 (6), highlighting the need to elucidate its pathogenesis and develop effective prevention and clinical management strategies.

Although local estrogen use is widely used to alleviate menopausal urogenital symptoms and improve vaginal mucosal status in postmenopausal women with POP, its effects remain incompletely understood. Epidemiological data link postmenopausal estrogen deficiency to higher POP risk (7), and local estrogen may modestly improve vaginal tissue thickness and matrix metabolism (8). However, randomized controlled trials show limited benefits for anatomical correction or long-term prolapse outcomes (9, 10). This disparity between basic research and clinical observation suggests an incomplete understanding of estrogen’s mechanisms of action within the complex *in vivo* tissue microenvironment. A key bottleneck lies in the inability of traditional methodologies, such as tissue homogenate analysis or *in vitro* cell models, to resolve the highly heterogeneous cellular composition, cell-specific responses, and spatial interaction networks within the primary POP target tissue—the vaginal wall (11–13).

The pelvic floor connective tissue constitutes a complex ecosystem comprising various cell types, including fibroblasts, immune cells, and vascular cells. Estrogen receptor (ER) expression varies significantly across these cell types (11), and the disrupted ERα/ERβ ratio observed in POP patients may predict differential responses to estrogen signaling among distinct cellular populations (11, 14). Fibroblasts, as the primary producers of the extracellular matrix (ECM) and core maintainers of tissue architecture, are considered important cellular targets through which estrogen may modulate connective tissue status. This cellular heterogeneity is a major contributor to the individual variation in response to estrogen use. Yet, a systematic characterization of the functional heterogeneity of fibroblasts, particularly within the postmenopausal POP vaginal wall at single-cell resolution, is currently lacking.

Furthermore, tissue function depends not only on cellular composition but also critically on its three-dimensional spatial architecture. Currently, knowledge regarding two pivotal questions remains limited: (1) How does estrogen influence the precise spatial localization of different cell types, especially functionally distinct fibroblast subpopulations, to modulate a reparative microenvironment? (2) How does estrogen remodel the short-range intercellular communication network to coordinate multicellular remodeling programs? A mechanistic understanding of these spatiotemporal dimensions may help address current limitations in clinical management.

Recent breakthroughs in single-cell RNA sequencing (scRNA-seq) and spatial transcriptomics have provided powerful tools for systematically parsing the cellular atlas, state transitions, spatial neighborhoods, and cell-cell communication within complex tissues at a systems level (13, 15). Notably, technologies like Visium HD, with their near-cellular spatial resolution (e.g., 2 μm), enable high precision in mapping the *in situ* distribution of cellular subpopulations, thereby revealing fine structural units within the microenvironment. Moreover, emerging computational pharmacology approaches can link single-cell transcriptomic data with drug sensitivity, allowing direct prediction of potential targeted strategies based on disease mechanisms and may facilitate translational development of more informed strategies. Therefore, this study aims to construct an integrated multi-omics atlas of the anterior vaginal wall in postmenopausal POP patients by leveraging 10x Genomics scRNA-seq and Visium HD spatial transcriptomics.

To achieve this, we profiled the anterior vaginal wall tissues from a cohort of postmenopausal POP patients, either untreated or treated with local estrogen. Our study aims to systematically characterize the cellular heterogeneity and functional states of the major cell types, with a particular focus on fibroblast subpopulations, at single-cell resolution. We further seek to delineate the specific regulatory impact of estrogen on the proportion, differentiation trajectory, and transcriptional activity of these fibroblast subsets. Moreover, we endeavor to elucidate how estrogen reshapes the spatial distribution of cells and reprograms the intercellular signaling network. Finally, by integrating computational pharmacology, we will predict the targetability of the identified repair programs, to provide a cellular and molecular framework for future tissue-directed strategies in POP care.

## 2 Results

### 2.1 Estrogen induces a selective remodeling of the vaginal wall cellular landscape in POP

**Figure 1:**
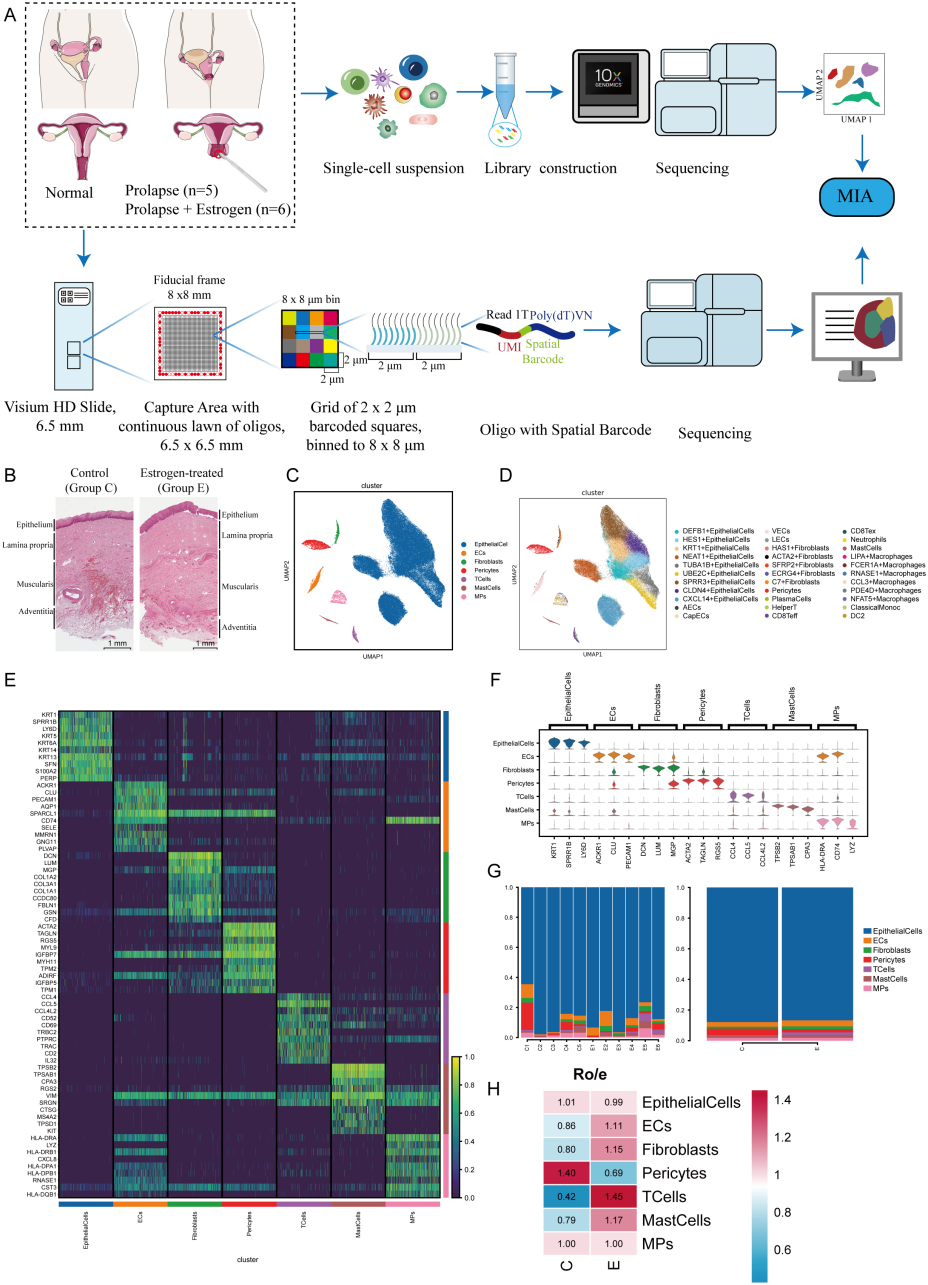
Construction of a multi-omics atlas of the postmenopausal POP vaginal wall and estrogen-mediated global remodeling of the cellular community. **(A)** Schematic overview of the experimental and analytical workflow for constructing the single-cell and spatial integrated atlas. Anterior vaginal wall tissues from postmenopausal POP patients, either untreated (Control, Group C, n=5) or treated with topical estrogen (Estrogen-treated, Group E, n=6), were subjected to scRNA-seq and Visium HD spatial transcriptomics, followed by multi-omics integration analysis. **(B)** Representative H&E staining of a full-thickness vaginal wall tissue section, confirming the preservation of tissue architecture. **(C)** UMAP projection of 116,775 high-quality cells from integrated scRNA-seq data, colored by the seven major cell types identified. **(D)** UMAP projection of cells following unsupervised sub-clustering of major cell types, revealing multiple functionally distinct cellular subpopulations, including epithelial, fibroblast, and immune cell subsets. **(E)** Heatmap displaying the expression of top canonical marker genes used for annotating the major cell types. **(F)** Violin plots showing the expression distribution of representative marker genes across the annotated cell types. **(G)** Stacked bar plot illustrating the global shifts in the relative proportions of major cell types between Group C and Group **(E, H)** Ro/e (Ratio of observed to expected) analysis quantifying the enrichment or depletion of specific cell types in response to estrogen treatment. An Ro/e >1 indicates enrichment, while <1 indicates depletion.

To systematically decipher the pathological microenvironment of postmenopausal pelvic organ prolapse (POP) and the tissue-level effects of topical estrogen at single-cell and spatial resolution, we performed 10x Genomics single-cell RNA sequencing (scRNA-seq) and Visium HD spatial transcriptomic sequencing on anterior vaginal wall tissues from POP patients. These included individuals either treated with topical estrogen (once daily for 6 weeks; E group, n=6) or left untreated (C group, control, n=5). Using Visium HD technology for *in situ* sequencing, we acquired spatial gene expression information by merging 2 x 2 μm barcoded spots into 8 x 8 μm analysis units. Subsequent multi-omics integration analysis (MIA) enabled the precise mapping of cell identities onto their tissue spatial locations, constructing a single-cell and spatial integrated atlas of the postmenopausal POP vaginal wall (**Figure 1A**). All enrolled POP patients were classified as POP-Q stage III–IV (**Table 1**). Histological examination (H&E staining) confirmed the acquisition of full-thickness vaginal wall structures (**Figure 1B**), allowing us to capture cellular dynamics before and after treatment.

**Table 1:** Baseline characteristics of the study cohort.

Group	POP (control group)					POP with Estrogen (estrogen-treated group)						P
Patient	P1	P2	P3	P4	P5	P6	P7	P8	P9	P10	P11	–
Age (years)	67	69	79	65	70	66	66	79	73	72	68	0.792
Height (m)	1.58	1.58	1.58	1.64	1.57	1.62	1.60	1.52	1.55	1.58	1.59	0.931
Weight (kg)	53	60	59	56	65	66	86	53	64	54	64	0.429
Body mass index (kg/m2)	21.23	24.03	23.63	20.82	26.37	25.15	33.59	22.94	26.64	21.63	25.32	0.247
Menopause Duration (years)	15	14	28	10	20	16	24	25	20	20	10	0.537
G (Gravidity)	4	5	5	5	4	6	4	5	6	4	4	0.792
P (Parity)	3	5	5	3	4	4	4	5	5	4	2	0.931
Hypertension	Yes	Yes	Yes	No	No	Yes	Yes	No	No	Yes	No	1.000
Diabetes	Yes	No	Yes	No	No	Yes	Yes	Yes	No	Yes	No	0.567

A total of 11 postmenopausal women with POP-Q stage III-IV were enrolled, including 5 in the control group (Group C) and 6 in the estrogen-treated group (Group E). Continuous variables (e.g., Age, BMI) were compared using the independent samples t-test. Categorical variables (e.g., Hypertension, Diabetes) were compared using the Chi-square test. All baseline characteristics showed no statistically significant differences between the two groups (*P* > 0.05), indicating that the groups were comparable.

Following stringent quality control, the scRNA-seq data yielded transcriptomes from 116,775 high-quality cells. Principal component analysis and UMAP clustering identified seven major cell types: epithelial cells, endothelial cells, fibroblasts, pericytes, T cells, mast cells, and mononuclear phagocytic cells (**Figure 1C**). Further subcluster analysis was performed to resolve finer heterogeneity (**Figure 1D**). The accuracy of cell type annotation was confirmed by differential expression gene analysis and the expression of canonical markers, such as high levels of COL1A1 in fibroblasts and PECAM1 in endothelial cells (**Figures 1E, F**; refer to **Supplementary Table 1** for a complete list of cell types and their marker genes).

Global cell proportion analysis revealed that estrogen treatment was associated with remodeling of the cellular community. Compared to the control group, the estrogen-treated group exhibited an increase in the relative proportions of fibroblasts and T cells, while the proportion of pericytes was significantly decreased (**Figure 1G**). This shift was quantitatively confirmed by Ro/e analysis: estrogen treatment was associated with enrichment of fibroblasts (Ro/e: 0.80 vs. 1.15) and T cells (Ro/e: 0.42 vs. 1.45), while reversing the enrichment of pericytes observed in the POP disease state (Ro/e: 1.40 vs. 0.69) (**Figure 1H**).

To further dissect cellular subpopulation heterogeneity, we performed subclustering analysis on endothelial cells, immune cells, and mononuclear phagocytes (**Supplementary Figures 1**-**4**). Endothelial cells were classified into multiple functional subsets, whose marker genes, ligand and receptor expression profiles are presented in **Supplementary Figure 1**. Immune cells were divided into four major subpopulations: plasma cells, helper T cells, effector CD8^+^ T cells and exhausted CD8^+^ T cells. Estrogen treatment markedly altered the composition and transcriptomic signatures of immune cell subsets. Gene Ontology (GO) and Kyoto Encyclopedia of Genes and Genomes (KEGG) enrichment analyses of differentially expressed genes indicated that estrogen regulates pathways involved in immune activation, apoptosis and inflammation (**Supplementary Figure 2**). Differential gene expression analysis of individual immune subsets revealed that estrogen broadly modulates genes related to immune cell activation, chemotaxis and cytokine secretion (**Supplementary Figure 3**). Mononuclear phagocytes consisted of neutrophils, macrophages, classical monocytes and dendritic cells, and estrogen remodeled pathways associated with inflammation, phagocytosis and metabolism in these cells (**Supplementary Figure 4**). Collectively, these findings demonstrate that estrogen exerts widespread regulatory effects across nearly all cell types in the vaginal wall microenvironment, reshaping not only stromal cells but also endothelial and immune cell networks.

These results suggest that estrogen does not uniformly affect all cell populations but rather specifically modulates the balance between stromal cells (e.g., fibroblasts and pericytes) and immune cells (e.g., T cells) (16, 17). The significant change in the abundance of fibroblasts, as the primary producers of the extracellular matrix, highlights their potential involvement in mediating estrogen-associated tissue effects (18). This finding provides a clear rationale for our subsequent in-depth investigation into fibroblast heterogeneity and functional reprogramming.

### 2.2 Estrogen drives a state-specific shift in fibroblasts toward a pro-repair phenotype

**Figure 2:**
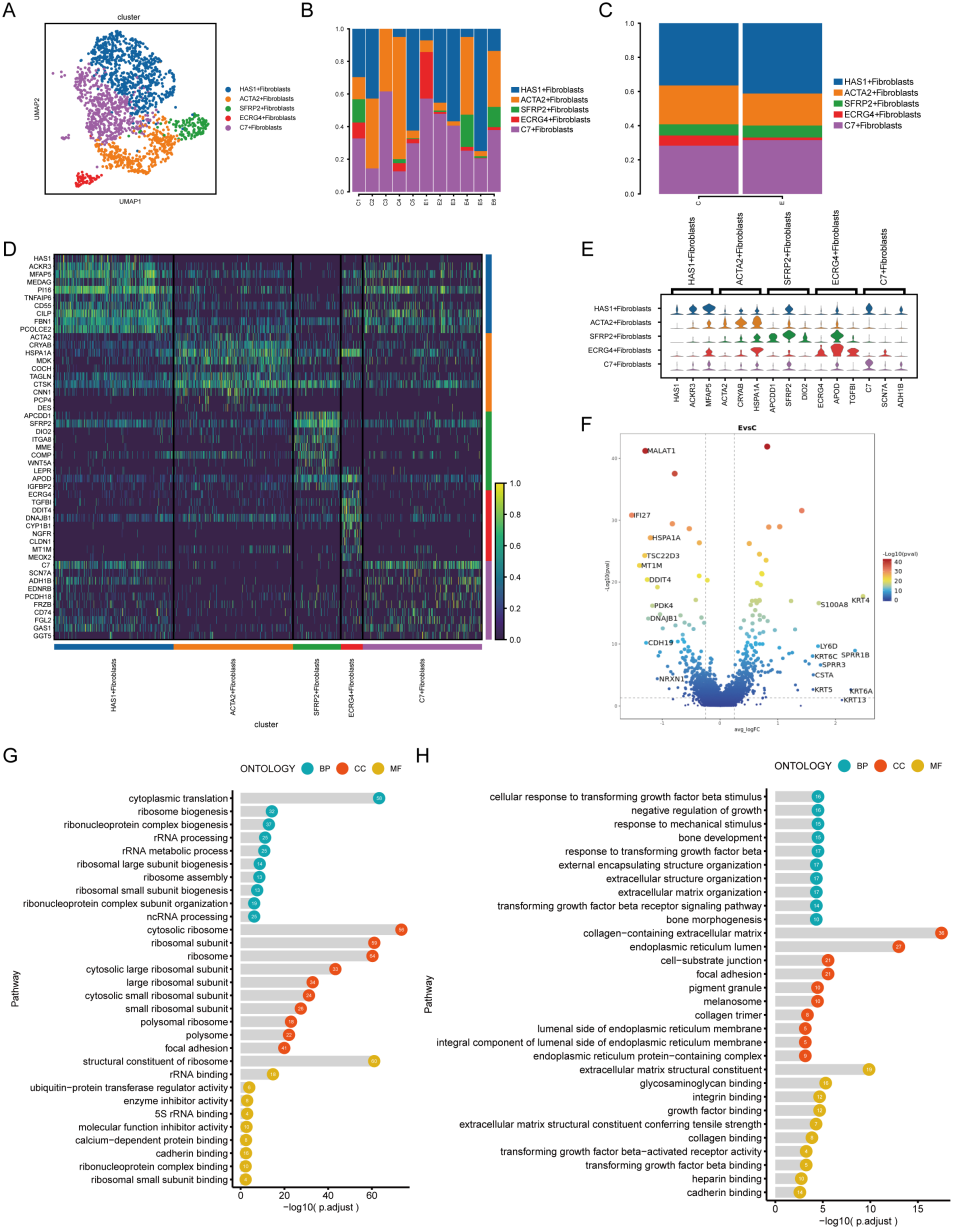
Estrogen reprograms fibroblast heterogeneity and induces a shift toward a pro-remodeling phenotype. **(A)** UMAP projection of fibroblasts from the integrated dataset, revealing five transcriptionally distinct subpopulations. **(B)** Stacked bar plot showing the relative abundance of each fibroblast subpopulation across all 11 individual samples. **(C)** Stacked bar plot depicting the composition of fibroblast subpopulations in control (Group C) and estrogen-treated (Group E) groups. **(D)** Heatmap of the top marker genes defining the five fibroblast subpopulations. **(E)** Violin plots showing the expression distribution of representative marker genes (e.g., HAS1, ACTA2) across the fibroblast subpopulations. **(F)** Volcano plot displaying the differentially expressed genes (DEGs) in fibroblasts after estrogen treatment. **(G)** Biological processes significantly enriched among the upregulated genes. **(H)** Biological processes significantly enriched among the downregulated genes.

Based on the observed overall expansion of fibroblasts, we hypothesized that estrogen’s effect might be subpopulation-specific. To test this, we performed unsupervised sub-clustering on fibroblasts, which successfully partitioned them into five transcriptionally distinct subpopulations (**Figure 2A**). Crucially, cell proportion analysis revealed that estrogen was not associated with uniform expansion of all subpopulations but exhibited selectivity: the proportion of the HAS1+ fibroblasts subpopulation was significantly increased post-treatment, whereas the proportion of the canonical myofibroblast subpopulation, ACTA2+ fibroblasts, relatively decreased (**Figures 2B, C**) (19, 20). This indicates that the estrogen-associated expansion of fibroblasts is associated with a selective shift in cell state prevalence.

To understand the functional implications of this proportional shift, we systematically annotated the functions of each subpopulation. Marker gene analysis showed that the HAS1+ fibroblasts subpopulation highly expresses hyaluronic acid synthase 1 (HAS1), identifying it as a primary cellular source for hyaluronic acid synthesis in the tissue. This suggests a potential role in improving tissue tension through enhanced matrix hydration and modulating the immune microenvironment (**Figures 2D, E**). In contrast, the ACTA2+ fibroblasts subpopulation exhibited typical myofibroblast characteristics, expressing α-smooth muscle actin (ACTA2), which is associated with tissue contraction and fibrosis. To quantitatively assess the functional differences among fibroblast subpopulations, we conducted functional scoring analysis based on signature gene sets (**Supplementary Figure 5**). The results showed that the ACTA2+ myofibroblast subset had high scores for profibrotic and contractile functions, whereas the HAS1+ fibroblast subset exhibited stronger anti-inflammatory and epithelial repair capacities. This estrogen-associated shift suggests that estrogen exposure is associated With the generation of a reparative matrix while concurrently suppressing aberrant fibrotic processes.

To further confirm this functional switch at the global transcriptome level, we analyzed differentially expressed genes (DEGs) in fibroblasts upon estrogen treatment. A volcano plot showed significant expression changes in 543 genes (**Figure 2F**). Gene Ontology (GO) pathway enrichment analysis revealed that upregulated genes were significantly enriched in protein synthesis pathways such as “cytoplasmic translation” and “ribosomal structural constituent” (**Figure 2G**). Conversely, downregulated genes were enriched in fibrosis-related pathways, including “cellular response to TGF-β stimulus” and “extracellular matrix structural constituent” (**Figure 2H**) (8). This opposing enrichment pattern is consistent with a shift in fibroblast functional state from “pro-fibrotic” to “pro-repair” by simultaneously activating protein anabolic metabolism and inhibiting TGF-β-driven fibrotic programs (19, 21).

### 2.3 Estrogen directly drives fibroblast differentiation toward a HAS1+ tissue-modulatory phenotype

**Figure 3:**
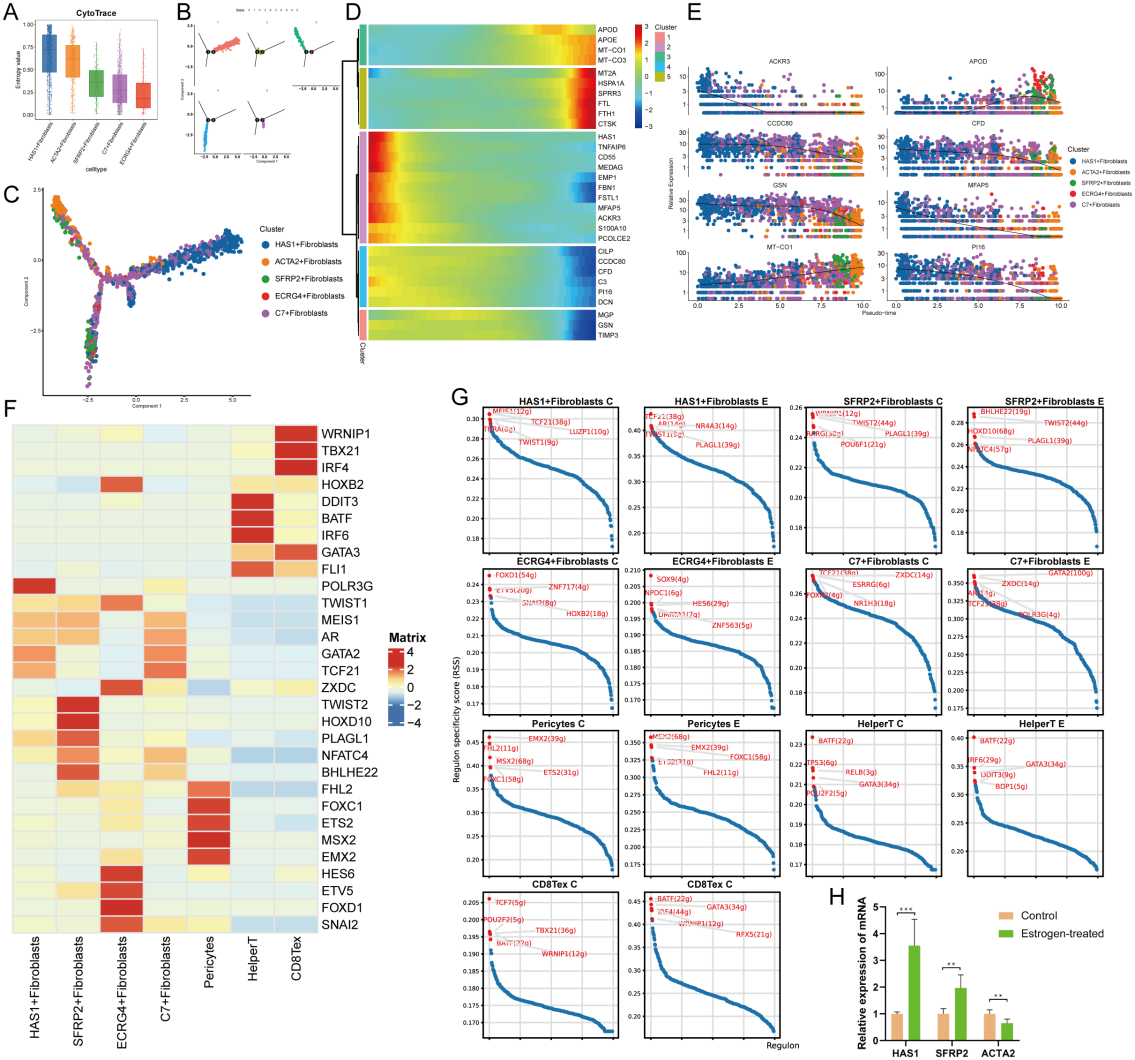
Estrogen guides fibroblast differentiation toward a pro-remodeling phenotype. **(A)** Box plot comparing the CytoTRACE-predicted differentiation states of fibroblasts between Group C and Group E, indicating that estrogen treatment maintains fibroblasts in a less differentiated, more plastic state. **(B)** Pseudotemporal trajectory of fibroblasts inferred by Monocle2, showing the transition paths from a central starting point to multiple distinct terminal states. **(C)** Distribution of cells from the five distinct states along the pseudotemporal trajectory, showing the specific enrichment patterns of cells from Group C and Group E across different states. **(D)** Heatmap of core gene clusters with expression patterns dynamically changing along the pseudotime trajectory. **(E)** Gene Ontology (GO) enrichment analysis of the trajectory-associated genes, revealing significant enrichment in pathways related to extracellular matrix organization and collagen metabolic process. **(F)** Transcription factor (TF) regulatory activity across fibroblast subpopulations analyzed by SCENIC, highlighting specific TF activation patterns. **(G)** Regulatory specificity scores of key TFs. **(H)** qPCR validation in primary human vaginal fibroblasts. Expression levels of HAS1, ACTA2, and SFRP2 in control vs. estrogen-treated groups.

To investigate whether the estrogen-associated tissue-modulatory phenotype is a stochastic event or a precisely regulated cell fate transition, we first assessed the developmental potential of fibroblasts. CytoTRACE analysis revealed that fibroblasts in the estrogen-treated group exhibited a significantly lower overall differentiation index compared to the control group, presenting a more juvenile and plastic state (**Figure 3A**). This suggests that estrogen may preserve progenitor-like characteristics, providing a cellular reservoir for differentiation toward a tissue-modulatory phenotype.

Subsequently, the pseudotemporal trajectory constructed by Monocle2 delineated the paths of cell state transition. Cells diverged from a relatively centralized starting point toward multiple functionally distinct terminal states (**Figure 3B**). Critically, cells from the estrogen-treated group were significantly enriched in the differentiation branch leading specifically to the reparative subpopulation HAS1+ fibroblasts (**Figure 3C**), suggesting that estrogen may preferentially support rather than passively select for this cell fate (22, 23).

To decipher the molecular programs occurring along this differentiation trajectory, we identified core gene clusters with dynamically changing expression (**Figure 3D**). These genes displayed clear temporal expression patterns: genes represented by ACKR3 and PI16 decreased along the trajectory, constituting a “stemness/plasticity maintenance” module; whereas genes represented by APOD and MT-CO1 increased, forming a “terminal functional execution” module. GO functional enrichment analysis of these trajectory-associated genes further revealed their significant enrichment in pathways directly related to tissue repair and remodeling, such as “extracellular matrix organization” and “collagen metabolic process” (**Figure 3E**). This suggests that the estrogen-associated differentiation pattern is functionally linked to tissue remodeling.

To identify upstream regulators, we performed SCENIC transcription factor network analysis, which uncovered key transcription factors with specific activity in HAS1+ fibroblasts (**Figures 3F, G**). Functionally, estrogen significantly increased the HAS1/ACTA2 ratio (~5.5-fold) and upregulated SFRP2 (~2.0-fold) (**Figure 3H**), confirming a shift from a pro-fibrotic to a tissue-modulatory phenotype.

### 2.4 Locating the tissue-modulatory unit: spatial transcriptomics reveals an estrogen-associated HAS1+ FIBROBLAST-PERICYTE NICHE

The transition in cell state must ultimately be located within the three-dimensional space of the tissue to fulfill its physiological function. To examine the fibroblast state transition revealed by single-cell analysis *in situ* and to investigate whether estrogen is associated with remodeling of cellular spatial organization, we integrated Visium HD spatial transcriptomic data.

**Figure 4:**
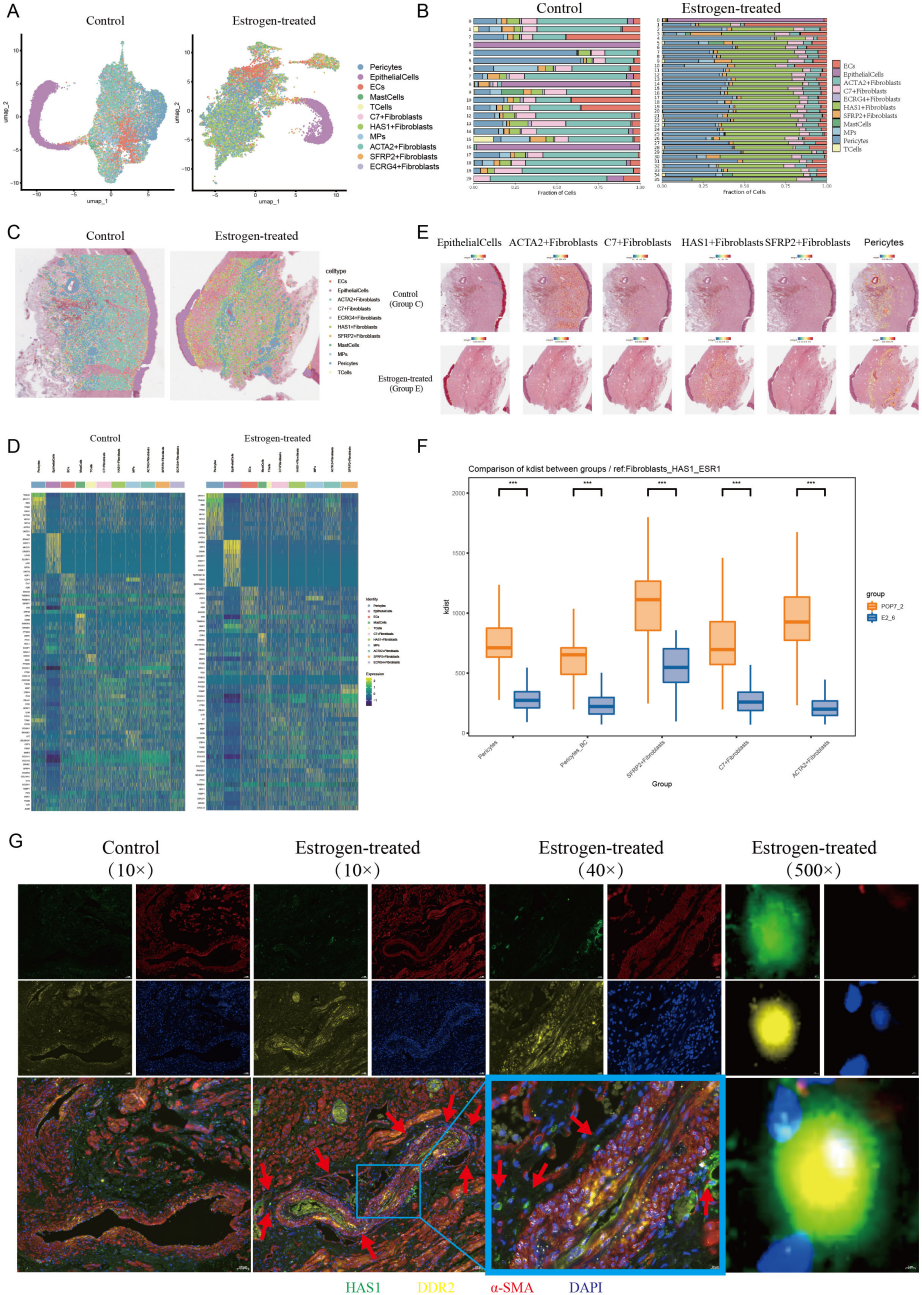
Spatial transcriptomics and immunofluorescence validation reveal an estrogen-induced HAS1+ fibroblast-pericyte tissue-modulatory niche. **(A)** Spatial clustering of vaginal wall tissues from representative control (Group C, left) and estrogen-treated (Group E, right) samples, showing distinct transcriptomic patterning induced by estrogen. **(B)** Quantitative analysis of cell type proportions deconvoluted from spatial transcriptomics data, confirming the increase in HAS1+ fibroblasts and decrease in C7+ fibroblasts after estrogen treatment. **(C)** Spatial distribution maps of key cell types. Estrogen induces co-localization of HAS1+ fibroblasts with pericytes, forming a structured niche, while contracting the distribution of ACTA2+ fibroblasts+ myofibroblasts. **(D)** Heatmaps of spatially variable genes in control and estrogen-treated tissues, showing cell-type-specific molecular reprogramming in the tissue-modulatory niche. A high-resolution optimized version is provided in **Supplementary Figure 6**. **(E)** Spatial weight analysis illustrating the specialized microenvironment constructed by HAS1+ fibroblasts, pericytes, mononuclear phagocytes (MPs), and T cells. **(F)** Spatial distances between HAS1+ESR1+ fibroblasts and neighboring cell types in control (POP) and estrogen-treated (E2) samples. Lower values indicate closer proximity. **(G)** Multiplex immunofluorescence (mIF) validation. Co-localization of HAS1 (green, matrix synthesis) and DDR2 (yellow, environmental sensing) signals around α-SMA+ (magenta, vasculature) structures in the estrogen-treated group, confirming the formation of a HAS1+DDR2+α-SMA- tissue-modulatory fibroblast population within a perivascular repair niche.

Unsupervised clustering of the spatial data first revealed that, in the analyzed samples, tissue regions from the estrogen-treated group exhibited a transcriptomically distinct clustering pattern compared to the control group (**Figure 4A**), This observation suggests that estrogen treatment may be associated with remodeling of the vaginal wall at both the transcriptional and spatial architectural levels, although these findings are derived from one representative sample per group and require validation in an independent cohort. Through spatial cell type deconvolution, we precisely mapped the *in situ* distribution of major cell types. Quantitative analysis indicated that the relative proportion of the reparative HAS1+ fibroblasts subpopulation significantly increased in the estrogen-treated group, while the proportion of the terminally differentiated C7+ fibroblasts subpopulation correspondingly decreased (**Figure 4B**), a finding consistent with our scRNA-seq results.

The key spatial evidence came from the precise localization of cellular subpopulations. In the estrogen-treated sample, we observed a notable spatial repositioning of the HAS1+ fibroblast subpopulation: compared with the control sample where these cells showed a relatively dispersed distribution, they exhibited a more aggregated pattern in regions highly enriched with pericytes, suggesting a potential spatial co-localization between the two (16, 24). Concurrently, the spatial distribution of the ACTA2-expressing myofibroblast subpopulation was contracted (**Figure 4C**). This spatial remodeling—”moving towards tissue-modulatory center and away from fibrotic areas”—may provide a physical foundation for functional cellular collaboration.

At the molecular level, our analysis of spatially variable genes suggested cell-type-specific molecular reprogramming induced by estrogen (**Figure 4D**; high-resolution version in **Supplementary Figure 6**). The expression signals of repair-related signature genes were significantly enhanced *in situ* in the HAS1+ fibroblasts subpopulation, while some pro-fibrotic genes in the ACTA2+ fibroblasts subpopulation showed a downregulation trend. Notably, pericytes spatially adjacent to HAS1+ fibroblasts also exhibited coordinated changes in their gene expression profiles, suggesting functional coupling achieved through spatial proximity. To further characterize the spatial microenvironment of this repair unit, we performed spatial neighborhood analysis. The results showed that the HAS1+ fibroblasts subpopulation exhibited stable spatial adjacencies with pericytes, mononuclear phagocytes, and T cells within the tissue (**Figure 4E**), collectively constituting a multicellular spatial niche.

To quantitatively assess the spatial organization of the repair niche, we calculated the spatial distances between HAS1+ESR1+ fibroblasts and neighboring cell populations, where lower values indicate closer proximity. As shown in **Figure 4F**, the distances between HAS1+ESR1+ fibroblasts and all analyzed cell types were consistently shorter in the estrogen-treated (E2) group compared to the control (POP) group, suggesting that estrogen is associated with closer spatial association. Notably, in the E2 group, HAS1+ESR1+ fibroblasts exhibited the closest proximity to ACTA2+ fibroblasts and pericytes, suggesting that these cell types are key components of the estrogen-associated repair niche. Based on the upper quartile (75%) of the distance distribution (threshold = 0.75), we identified a pericyte subpopulation with high spatial sensitivity to HAS1+ESR1+ fibroblasts.

Based on the functional heterogeneity of fibroblasts and the potential for a repair phenotype switch suggested by our prior single-cell and spatial transcriptomic analyses, we hypothesized that estrogen induces a reparative fibroblast subpopulation possessing dual functional capabilities: “matrix synthesis” and “environmental sensing.” To test this, we designed a multiplex immunofluorescence (mIF) experiment targeting the key hyaluronic acid synthase HAS1, the collagen receptor DDR2, and the myofibroblast marker α-SMA.

The multiplex immunofluorescence (mIF) results were consistent with our hypothesis and suggested a key spatial restructuring phenomenon. In the estrogen-treated group, red arrows clearly indicate that HAS1^+^ fibroblasts closely adjoin the luminal borders of α-SMA^+^ vascular structures (**Figure 4G**), while this region is also enriched with DDR2 signal (yellow). This indicates that estrogen-induced HAS1^+^DDR2^+^ reparative fibroblasts are not randomly distributed but are specifically recruited and tightly clustered around vascular smooth muscle cell (α-SMA^+^)-constituted perivascular zones. This area is a known hotspot for intercellular interaction and signal exchange. The phenomenon suggests that estrogen is associated with perivascular microenvironment changes and may guide reparative cells to migrate toward and accumulate at this specific anatomical site, thereby morphologically and spatially constructing a stem cell niche-like “perivascular tissue-modulatory niche.” Within this unit, the closely vascular-adjoined HAS1^+^DDR2^+^ fibroblasts can simultaneously utilize nutritional support from the vasculature and, through DDR2 as a key sensor of the collagen microenvironment, couple the chemotactic behavior of HAS1^+^ cells with matrix synthesis, forming an integrated structure–function remodeling event. This provides *in situ* protein-level support for the collaborative behavior of the “fibroblast–pericyte axis” in pelvic organ prolapse tissue remodeling, at least in the samples examined. Functionally, this spatially organized structure resembles stem cell microenvironments reported in various tissues, offering potential spatial anchoring and signaling support for repair cells (24).

Furthermore, in the estrogen-treated group, we successfully identified a cell population matching the predicted profile: they concurrently highly expressed HAS1 and DDR2 but did not express α-SMA (**Figure 4G**). This phenotypic signature provided protein-level evidence supporting the existence of the hypothesized “functionally coupled repair cell.” This cell type not only possesses the ability to construct a reparative matrix via hyaluronic acid synthesis but can also sense collagen microenvironment signals through DDR2, enabling dynamic regulation of its functional state. Their α-SMA-negative character further suggests that this population has not differentiated into pro-fibrotic myofibroblasts, thereby consistent with a remodeling process oriented toward functional tissue modulation rather than pathological fibrosis.

By integrating spatial transcriptomics with *in situ* immunofluorescence validation, this study provides evidence suggesting that estrogen is associated with not only reprogramming of fibroblast states at the single-cell level but also the formation of a structured repair niche involving HAS1+ fibroblasts and pericytes within the tissue space. This discovery organically links cell state transition with spatial restructuring, provides spatial biological insights for understanding estrogen-mediated microenvironment remodeling.

### 2.5 Estrogen is associated with intercellular communication to activate a fibroblast-pericyte signaling axis

**Figure 5:**
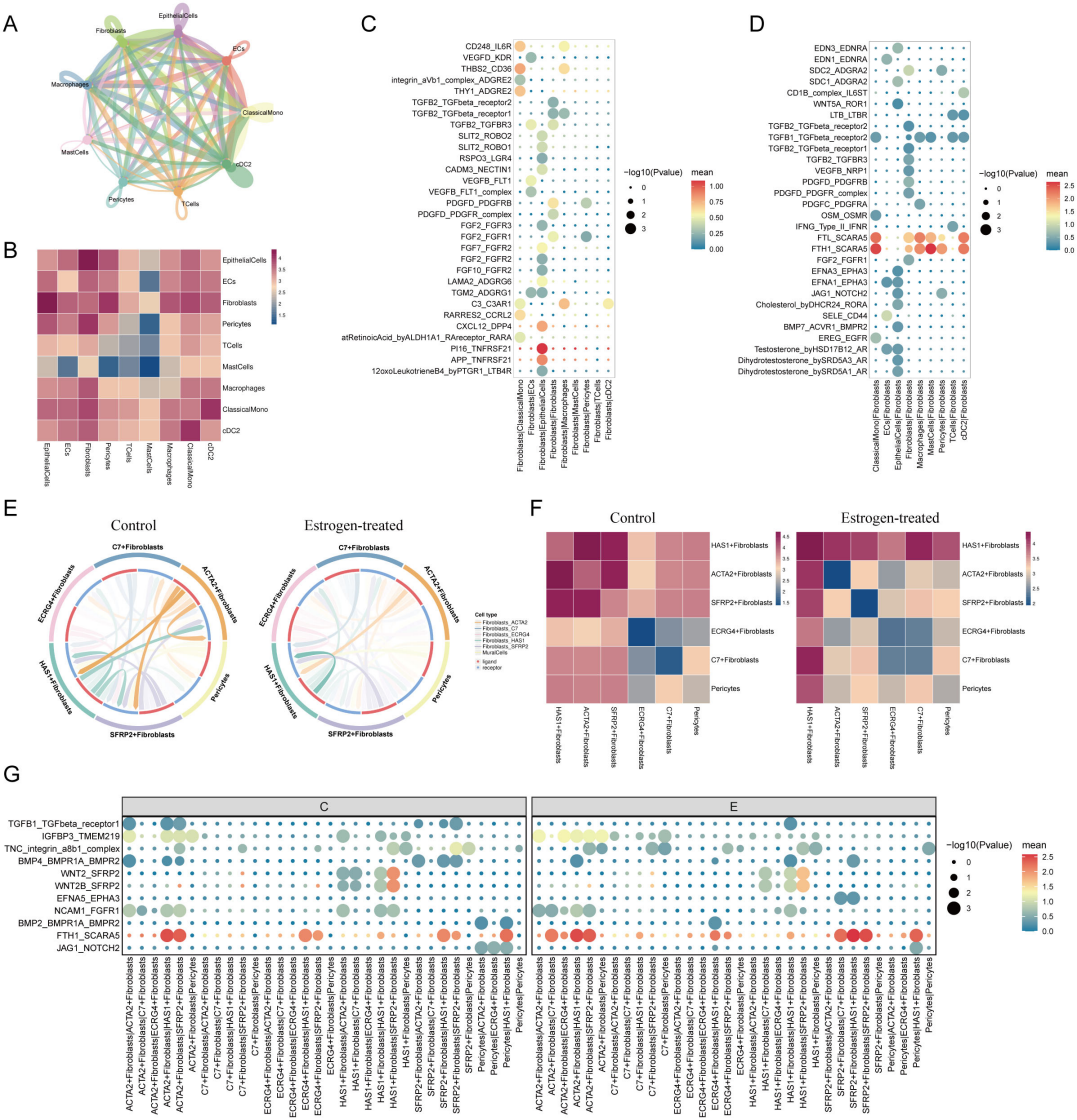
Estrogen activates a fibroblast-pericyte signaling axis by remodeling intercellular communication. **(A)** Circle plot visualizing the global cell-cell communication network among major cell types, demonstrating fibroblasts as a central signaling hub. **(B)** Heatmap summarizing the number of significant ligand-receptor interactions between different cell type pairs. **(C, D)** Functional characterization of fibroblast signaling capabilities. **(C)** Dot plot showing the top ligands significantly expressed by fibroblast subpopulations. **(D)** Dot plot showing the top receptors significantly expressed by fibroblast subpopulations. **(E)** Chord diagrams comparing the specific ligand-receptor interactions between fibroblast subpopulations and Pericytes (including pericytes) in control (left) and estrogen-treated (right) conditions. **(F)** Heatmaps comparing the interaction strength between fibroblast subpopulations and Pericytes in control (left) and estrogen-treated (right) groups, showing enhanced HAS1+ fibroblasts-pericyte interactions. **(G)** Dot plot analyzing the expression of key ligand-receptor pairs (including TGFB1-TGFBR1 and FTH1-SCARA5) between fibroblast subpopulations and Pericytes across experimental groups.

Building upon the spatial adjacency revealed by spatial transcriptomics, we next investigated whether this physical proximity translates into functional molecular crosstalk that coordinates the execution of the tissue remodeling program. Through systematic cell-cell communication analysis, we found that fibroblasts appear as a prominent signaling node within the cellular interaction network of the vaginal wall. Visualization analysis revealed dense and complex connection networks formed between various fibroblast subpopulations and pericytes, epithelial cells, and immune cells (**Figures 5A, B**).

In-depth analysis of the functional properties of fibroblasts within this signaling network identified a dual role: they act as active signal senders, expressing various ligands (e.g., PI16, CXCL12) to transmit regulatory cues to neighboring cells, and as signal receivers, sensing microenvironmental signals through a rich repertoire of receptors (e.g., FTL, FTH1) (**Figures 5C, D**). Network centrality analysis further indicated that the signaling axis formed by fibroblasts and pericytes was prominent within the overall communication network, suggesting that this cellular pair may represent an important signaling module involved in tissue homeostasis.

To quantify the impact of estrogen on specific cellular interactions, we performed a detailed ligand-receptor pair analysis. Although the Ro/e analysis indicated an overall decrease in the proportion of pericytes, we observed that in the estrogen-treated group, the number of interactions between HAS1+ fibroblasts and pericytes increased by approximately 25%, with a trend toward enhanced interaction strength (**Figures 5E, F**; **Supplementary Table 4**) (19, 25). This finding correlates with the observed increase in their spatial proximity.

Further molecular mechanism dissection revealed key signaling pathways mediating this enhanced interaction. We identified several specifically regulated ligand-receptor pairs, including TGFB1-TGFBR1, FTH1-SCARA5, among others (**Figure 5G**) (26). Among these, the regulation of the TGF-β signaling pathway may influence the cellular fibrotic phenotype and the balance of tissue repair, while the interaction between FTH1 (ferritin heavy chain) and SCARA5 (scavenger receptor class A member 5) may be involved in iron metabolism homeostasis and cytoprotective processes. Such coordinated signaling changes are consistent with the established multidimensional regulatory role of estrogen signaling in pelvic floor connective tissue (27). Meanwhile, we analyzed estrogen-induced transcriptomic alterations in pericytes (**Supplementary Figure 7**). Functional enrichment analyses showed that estrogen regulates multiple biological processes including metabolism, protein synthesis, cell adhesion, ECM remodeling and immune responses in pericytes. Such functional reprogramming, together with enhanced fibroblast-pericyte crosstalk, supports the function of the perivascular reparative niche.

### 2.6 Toward precision modulation: computational pharmacology suggests the drug targeting potential of the tissue-modulatory unit

Based on our preceding findings—that estrogen is associated with fibroblast differentiation into a HAS1+ tissue-modulatory phenotype, is associated with its spatial positioning, and is associated with niche formation with pericytes—we posed a critical question: Does this highly coordinated program of cellular and spatial remodeling possess druggable potential? To address this, we utilized the BeyondCell computational platform to predict drug sensitivity based on our single-cell transcriptomic data.

**Figure 6:**
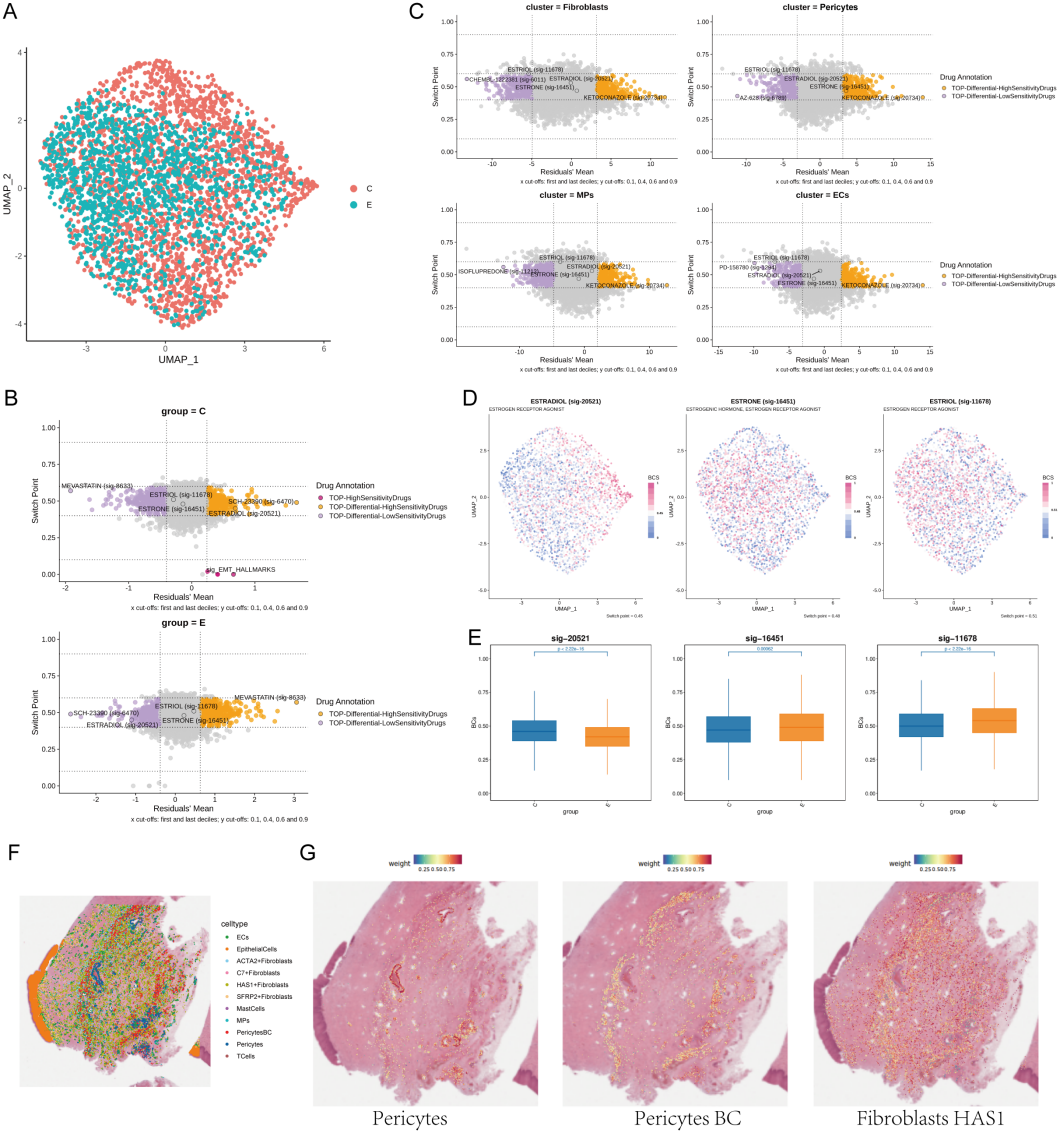
Computational pharmacology suggests the drug targeting potential of the estrogen-induced tissue-modulatory unit. **(A)** UMAP visualization of the global drug sensitivity space, showing distinct distribution patterns between cells from Group C (control) and Group E (estrogen-treated). **(B)** Drug sensitivity profiles of major cell types (fibroblasts, pericytes, mononuclear phagocytes, and endothelial cells) to various pharmacological agents. **(C)** Sensitivity patterns of key cell types to natural estrogen subtypes (estrone, estradiol, estriol) in Group C (left) and Group E (right), showing cell type-specific responses. **(D)** UMAP distribution of biosensitivity scores (BCS) for individual natural estrogen subtypes across all cells, demonstrating differential sensitivity patterns. **(E)** Quantitative comparison of BCS for the three natural estrogen subtypes between Group C and Group E, confirming significant changes in sensitivity profiles. **(F)** Spatial validation of the tissue-modulatory unit, showing co-localization of HAS1+ fibroblasts (red) and pericytes (blue) in estrogen-treated tissues. **(G)** Spatial weight analysis mapping the precise anatomical distribution of pericytes and HAS1+ fibroblasts within the vascular niche.

Initially, in the global drug sensitivity space, cells from the control and estrogen-treated groups exhibited distinct distribution patterns (**Figure 6A**). This finding suggests that the estrogen-modulated cellular microenvironment exhibits a distinct transcriptomic pattern and associated pharmacological profile, theoretically enabling targeted intervention.

In-depth analysis of the global drug sensitivity patterns (**Figure 6B**) revealed that estrogen treatment reshaped the cellular sensitivity profiles toward non-estrogen drugs. Crucially, however, cells in both treated and control groups consistently exhibited “low sensitivity” toward the three natural estrogens (estriol, estrone, and estradiol). This phenomenon suggests that the overall tissue-modulatory effect of estrogen is not achieved through transient, potent agonism of intracellular estrogen receptor signaling. Instead, it likely involves inducing a sustained, programmed remodeling of cell states and the microenvironment, whereby cells, after completing reprogramming, no longer exhibit high sensitivity to additional estrogen stimulation. This may represent a hallmark of tissue-remodeling program completion and the establishment of a new homeostasis.

Focusing further on fibroblasts and pericytes, which were identified as core targets of estrogen regulation, we analyzed the cell-specific action patterns of different estrogen subtypes. In fibroblasts, all three natural estrogens showed low sensitivity, consistent with a mechanism where estrogen primarily induces state transition rather than direct, strong activation. Notably, in pericytes, estrone was predicted to show clear high sensitivity (**Figure 6C**). This observation suggests that the overall tissue-modulatory influences may stem from the synergistic action of different subtypes on distinct cell populations: estradiol may primarily drive fibroblast state reprogramming, whereas estrone, by potentially acting on pericytes, may indirectly contribute to the cooperative interactions of the fibroblast-pericyte axis.

Further dissection of the sensitivity distribution patterns for different estrogen subtypes at single-cell resolution revealed that the biosensitivity score (BCS) distribution of estradiol exhibited unique characteristics: high BCS values were primarily enriched in control group cells, while low BCS values were significantly enriched in estrogen-treated group cells (**Figure 6D**). Quantitative analysis indicated that compared to the control group, the BCS score for estradiol significantly decreased in the treated group, whereas the BCS scores for estrone and estriol showed opposite trends (**Figure 6E**). This differential pharmacological signature is consistent with differential actions of estrogen subtypes in the tissue remodeling program (28).

To examine the biological basis of the computational pharmacology predictions, we analyzed the key cell populations targeted by the sensitivity predictions via spatial transcriptomics. Spatial cell localization analysis showed the clear co-localization of HAS1+ fibroblasts and pericytes in estrogen-treated tissues (**Figure 6F**). In-depth spatial weight analysis further revealed the specialized spatial structures constructed by these cell populations during the remodeling process (**Figure 6G**), where pericytes exhibited focal enrichment around tubular structures, and HAS1+ fibroblasts displayed a distribution pattern highly coordinated with that of pericytes.

Together, these findings support that estrogen-associated tissue effects are linked to the formation of a structured “HAS1+ fibroblast-pericyte” spatial unit. Furthermore, the computational pharmacology analysis generates the hypothesis that this tissue-modulatory unit may possess differential sensitivity to distinct estrogen subtypes. However, these predictions are derived purely from transcriptomic signatures and await experimental validation. Therefore, they should be considered hypothesis-generating for future mechanistic and translational studies, rather than providing immediate therapeutic guidance.

## 3 Discussion

Pelvic organ prolapse (POP), a highly prevalent pelvic floor disorder, has long faced challenges in clinical management due to inconsistent tissue-modulatory outcomes. Although epidemiological evidence supports an association between the postmenopausal low-estrogen state and increased POP risk (7, 29), local estrogen is widely used to improve vaginal mucosal status and urogenital symptoms, randomized controlled trials (RCTs) reveal considerable variability in its efficacy for improving patients’ subjective symptoms and achieving long-term anatomical restoration, presenting a notable “double-edged sword” profile (9, 10, 30, 31). This disparity between basic research and clinical observation indicates persistent gaps in our understanding of estrogen’s mechanisms of action within the complex, heterogeneous tissue microenvironment.

Traditional research has largely relied on molecular biological analyses of tissue homogenates or simplified *in vitro* cell models (32, 33). While these approaches have successfully uncovered estrogen’s effects on macroscopic processes such as collagen metabolism and fibroblast proliferation (18, 34, 35), their fundamental limitation lies in the inability to resolve cellular heterogeneity within the tissue and their neglect of the three-dimensional spatial architecture essential for intercellular communication. Consequently, while we can observe the “net effect” following estrogen treatment, we remain unable to determine which specific cell populations execute these effects, what state transitions these populations undergo, or how they are spatially organized to coordinately achieve tissue-remodeling functions.

The advent of single-cell transcriptome sequencing has recently provided new perspectives for analyzing tissue heterogeneity. Studies have identified significant individual variations in estrogen receptor expression in the pelvic floor tissues of POP patients (11, 14), as well as tissue-specific alterations in membrane receptors such as GPER (36, 37). More importantly, scRNA-seq studies have identified functionally distinct fibroblast subpopulations within the vaginal wall (13), while also revealing that infiltrating immune cells and their different polarization states in pelvic floor tissues are closely associated with estrogen regulation and tissue remodeling outcomes (16, 38). These findings highlight the limitations of traditional two-dimensional culture models in recapitulating complex cellular interactions (39).

Building on this knowledge, our study employed single-cell RNA sequencing and Visium HD spatial transcriptomics to overcome the limitations of traditional approaches (32, 33, 39), representing a conceptual shift from “holistic observation” to “programmatic dissection.” We not only systematically defined the cellular composition of the vaginal wall microenvironment but also precisely mapped their functional states (12, 13, 40), differentiation trajectories, spatial coordinates, and communication networks (16). Through systematic multi-omics integration, our findings support a mechanistic model: estrogen exerts tissue-modulating effects not by broadly stimulating tissue regeneration (18, 35), but by executing a set of precisely coordinated cellular programs—reprogramming key cell states (19), reconstructing functional spatial niches at specific anatomical sites, and rewiring intercellular communication (15, 16)—thereby guiding the disordered pathological microenvironment into an orderly remodeling program.

### 3.1 Functional Heterogeneity and State Reprogramming of Fibroblasts

Within the pelvic floor connective tissue, fibroblasts, as the predominant cellular component and primary producers of the extracellular matrix (ECM), have long been a central focus in understanding POP pathophysiology (40). However, a fundamental limitation of previous research has been the treatment of fibroblasts as a functionally homogeneous population that becomes uniformly activated in the pathological state. Substantial evidence indicates an increase in fibroblasts exhibiting myofibroblast characteristics in the pelvic support tissues of POP patients (19), accompanied by aberrant activation of the TGF-β signaling pathway (26, 41, 42) and disordered collagen metabolism (32, 43, 44). While this “pro-fibrotic” perspective partially explains the phenomenon of ECM remodeling imbalance in POP tissues, it creates a cognitive paradox: if estrogen merely acts as a simple stimulator of fibroblast proliferation (18, 35), it should logically exacerbate, rather than ameliorate, this fibrotic tendency.

Our scRNA-seq analysis revealed the “heterogeneous veil” of fibroblasts in the POP vaginal wall, systematically identifying five transcriptionally distinct fibroblast subpopulations with divergent functional orientations. By comparing estrogen-treated and untreated groups, we found that estrogen does not function as an indiscriminate “global activator” (18, 35), but rather as a precise “cell state selector.” Specifically, estrogen was associated with selective enrichment of the HAS1+ fibroblasts subpopulation, characterized by high expression of hyaluronic acid synthase 1, while relatively suppressing the canonical, ACTA2-rich myofibroblast subpopulation (19). This finding is significant because hyaluronic acid, as a crucial ECM component, enhances tissue hydration, modulates immune responses, and promotes tissue homeostasis—properties starkly contrasting with the mere collagen deposition that leads to fibrosis (43, 44). Consequently, our study revises the simplistic view that “fibroblasts solely play a pro-fibrotic role in POP” (32, 40), revealing the existence of functionally antagonistic subpopulations within them and, for the first time, identifying the HAS1+ fibroblasts subpopulation as a key cellular executor of estrogen-mediated tissue remodeling.

To delve deeper into the mechanism of this estrogen-guided cell state conversion, CytoTRACE analysis revealed that fibroblasts in the estrogen-treated group overall maintained greater developmental potential and plasticity. The pseudotemporal trajectory constructed by Monocle2 further delineated paths differentiating from a relatively primitive state towards multiple terminal subpopulations. Cells from the estrogen-treated group were significantly enriched in the branch leading to HAS1+ fibroblasts, suggesting that estrogen’s role is to actively guide cell fate rather than passively select for pre-existing states. SCENIC transcription factor network analysis further identified key regulatory factors with specific activity in the HAS1+ subpopulation. These findings collectively outline a regulatory program by which estrogen directs multipotent fibroblast precursors toward a tissue-modulatory phenotype (21–23).

### 3.2 Construction and functional specialization of the spatial niche

While single-cell transcriptomics has revealed cellular heterogeneity (13), a fundamental question remains unresolved: Where are these transcriptionally defined, tissue-remodeling-potent cell subpopulations precisely located within the complex tissue architecture? The current understanding of POP clinical management mechanisms is almost entirely built upon the dimension of “altered cellular composition and state” (18, 19, 35), lacking spatial validation. This gap prevents us from answering where precisely tissue-remodeling events occur within the tissue and from understanding how different cell types couple physically and functionally through spatial proximity (16).

Our study, by integrating Visium HD spatial transcriptomics with multiplex immunofluorescence staining, successfully bridged this gap, linking the cell state changes revealed by single-cell analysis to their precise spatial localization within the tissue. Unsupervised clustering of the spatial transcriptomic data showed a spatial restructuring of the overall transcriptional state in estrogen-treated tissues. Through cell type deconvolution, we observed that in the estrogen-treated sample, the tissue-modulatory HAS1+ fibroblasts subpopulation did not appear uniformly distributed but showed greater proximity to perivascular regions enriched with pericytes, suggesting a tight spatial association between the two (16). Concurrently, the spatial distribution of the pro-fibrotic ACTA2+ fibroblasts subpopulation was relatively contracted (19). This dynamic spatial repositioning—”selective aggregation and concomitant contraction”—is consistent with the possibility that estrogen is associated with reconfiguration of cellular spatial distribution, potentially forming a specialized “perivascular tissue-modulatory niche” *in situ*.

To dissect the cellular and molecular basis of this niche, we performed *in situ* validation at the protein level. Immunofluorescence results indicated that the cells specifically enriched around vasculature post-estrogen treatment were a population concurrently expressing high levels of HAS1 and the collagen receptor DDR2, but lacking expression of α-SMA. This unique HAS1+DDR2+α-SMA- phenotype is consistent with a novel class of tissue-modulatory cells functionally equipped with dual capabilities—”matrix synthesis” and “environmental sensing”—while successfully avoiding the “fibrotic” terminal fate. Specifically, HAS1 empowers these cells to synthesize hyaluronic acid, constructing a hydrated, elastic regulatory matrix to improve tissue tension. DDR2, as a key collagen receptor, enables them to continuously sense mechanical and chemical signals from the surrounding collagen microenvironment, facilitating dynamic dialogue between the cell and the ECM. The α-SMA- phenotype clearly distinguishes them from terminally differentiated, contractile, tissue-hardening-driving myofibroblasts (19). The combination of these three characteristics allows this cell type to achieve a complete *in situ* “sensing-response” functional loop at the perivascular hub, a nexus for nutrient and signal exchange.

### 3.3 Remodeling of the cellular communication network and coordinated regulation

While physical proximity between cells is a necessary condition for functional collaboration, it is not sufficient. We therefore investigated whether the estrogen-guided spatial co-localization translates into functional molecular crosstalk that coordinates the execution of the tissue-remodeling program. Previous research into the molecular mechanisms of POP, including extensive reports on the aberrant activation of classic signaling pathways such as transforming growth factor-β (TGF-β) (26, 41, 42), has largely operated under an implicit assumption: that activity changes in these pathways are primarily cell-autonomous behaviors. For instance, studies have predominantly focused on expression changes of TGF-β receptors or downstream SMAD proteins within fibroblasts themselves (19, 45). This “cell-centric” perspective largely overlooks the crucial role of intercellular communication as an upstream regulator of signaling pathways.

Our cell-cell communication analysis addressed this limitation, shifting the mechanistic understanding from the intracellular to the intercellular realm. The results suggest that fibroblasts act as a prominent signaling node of the signaling network within the vaginal wall tissue (40). Importantly, we found that estrogen’s regulation of key signaling pathways is achieved by remodeling the communication relationships between specific cell pairs. Taking the canonical TGF-β pathway as an example, although the overall proportion of pericytes decreased after estrogen treatment, the number and strength of interactions between the tissue-modulatory HAS1+ fibroblasts subpopulation and pericytes showed a marked increase by approximately 25%. This implies that estrogen does not simply increase or decrease the overall “volume” of TGF-β signaling across the tissue. Instead, akin to adjusting the “bandwidth” of a communication network, it appears to selectively modulate signal flux through the particular “communication channel” of the “fibroblast-pericyte” pair. This aligns with the broader paradigm that estrogen exerts tissue-modulating effects by reprogramming intercellular communication networks, a core aspect of its multidimensional regulation of tissue homeostasis (27).

Further molecular mechanism dissection through ligand-receptor pair analysis not only confirmed the specific regulation of known pathways, such as TGFB1-TGFBR1, within specific cell pairs but also identified a series of novel ligand-receptor pairs, like FTH1-SCARA5, previously underappreciated in the POP field. The interaction between FTH1, a key protein for iron storage and antioxidant stress, and the scavenger receptor SCARA5 suggests that iron metabolism homeostasis and cytoprotective mechanisms may be as integral to maintaining the function of the tissue-modulatory niche as the ECM remodeling we previously focused on (46). The discovery of these non-canonical pathways indicates that the stability of the estrogen-constructed tissue-modulatory niche is supported by a diversified signaling network.

### 3.4 Clinical management paradigm shift and translational prospects

Building upon these discoveries, we face a critical translational question: how can these mechanistic insights be converted into actionable clinical management and tissue-modulating strategies? Traditional POP drug development has long focused on the single dimension of “pathology suppression,” for instance, by striving to develop TGF-β signaling inhibitors or matrix metalloproteinase inhibitors to block excessive ECM degradation or abnormal deposition (26, 34, 41). However, such “anti-fibrotic” or “anti-catabolic” strategies have repeatedly encountered setbacks in clinical development. The fundamental reason lies in their sole aim to “block the bad” while failing to “promote the good” (20, 47)—that is, they do not actively activate and support the endogenous, orderly tissue-remodeling programs within the tissue.

Our BeyondCell computational pharmacology analysis offers a new perspective on this challenge. The analysis revealed that the cell microenvironment remodeled by estrogen exhibits a distribution in the global drug sensitivity space that is distinct from the untreated group, implying it displays a distinct pharmacological responsiveness profile. At single-cell resolution, different natural estrogen subtypes showed differential predicted sensitivities towards the distinct cell types constituting the tissue-modulatory unit: fibroblasts, which play a central role in driving remodeling, exhibited low sensitivity to estradiol, estrone, and estriol, consistent with a model in which estrogen is primarily linked to cell state reprogramming, rather than direct potent proliferative activation (19). In contrast, pericytes, acting as “collaborative partners” within the tissue-modulatory niche, displayed clear high sensitivity to estrone.

This observation suggests that estrogen’s tissue-modulating influences may stem from the synergistic action of different subtypes on different cell populations: estradiol likely serves as the primary “instructional signal” initiating fibroblast reprogramming (22, 48), whereas estrone, by acting directly on pericytes, indirectly consolidates and supports the cooperative interactions and niche stability of the “fibroblast-pericyte axis”. This cell type-specific pharmacological profile not only provides a fresh perspective for understanding the complex actions of estrogen but also provides theoretical support that this HAS1+ fibroblast–pericyte unit may serve as a plausible framework for future targeted research (23).

Based on this framework, future clinical and tissue-modulating strategies need no longer be confined to broad-spectrum hormone replacement or single-pathway inhibition. We can envision more precise interventions, including: developing small molecules that promote the tissue-modulatory HAS1+ fibroblast phenotype; utilizing biomaterial or chemokine strategies to mimic or enhance the “homing” of HAS1+DDR2+ tissue-modulatory cells to the perivascular niche (49, 50); and designing combination regimens incorporating different estrogen subtypes or specific receptor modulators based on cell-specific pharmacological profiles (28).

### 3.5 Study limitations

Several limitations of this study should be acknowledged. First, although CellChat analysis predicted enhanced fibroblast–pericyte communication via ligand–receptor pairs such as TGFB1–TGFBR1 and FTH1–SCARA5, these predictions are inferred solely from transcriptomic data and require further experimental validation. Since most predicted pairs involve secreted ligands, conventional co-immunofluorescence is technically limited by high false-negative rates; methods including proximity ligation assay (PLA) and conditional knockout models are better suited for future mechanistic verification.

Second, it remains challenging to accurately discriminate fibroblast subtypes from other stromal cell populations in vaginal wall tissue relying merely on conventional immunofluorescence, due to the generally low specificity of common mesenchymal markers. In general, genes with extremely low transcriptional expression rarely produce detectable protein signals under standard immunofluorescence conditions. In our scRNA-seq data, HAS1 shows negligible expression in all cell lineages other than fibroblasts. Accordingly, HAS1-positive immunofluorescence signals can be reliably assigned to the annotated HAS1^+^ population with typical fibroblast molecular features.

Third, spatial transcriptomics was performed on one sample per group (n=1 for control, n=1 for estrogen-treated). This limited sample size precludes statistical generalization of the spatial niche findings, including the observed co-localization of HAS1+ fibroblasts with pericytes. Therefore, the spatial conclusions presented in this study should be considered exploratory and hypothesis-generating rather than definitive. To mitigate this limitation, we performed orthogonal validation using multiplex immunofluorescence (mIF) on multiple independent samples from each group, which supported the key spatial observations. Nevertheless, expanded independent spatial transcriptomics cohorts are essential in future work to validate the existence and functional significance of the perivascular reparative niche. Additionally, the lack of healthy postmenopausal vaginal wall tissue as an independent reference limits our ability to fully distinguish disease-specific alterations from general estrogen-related physiological changes.

Fourth, the computational pharmacology predictions of cell-type-specific estrogen subtype sensitivity are purely in silico and derived from transcriptomic signatures using the BeyondCell platform. These predictions have not been validated by *in vitro* or *in vivo* experiments, such as primary cell cultures, functional assays, or animal models. Therefore, any translational claims regarding differential therapeutic targeting of specific cell populations remain speculative at this stage. Future studies using isolated primary fibroblasts and pericytes treated with distinct estrogen subtypes are essential to functionally validate these predictions.

Nevertheless, the primary goal of this study is to provide a high-quality, publicly available resource of scRNA-seq and Visium HD spatial transcriptomic data, to facilitate further research on pelvic organ prolapse pathogenesis and estrogen-mediated vaginal tissue remodeling.

### 3.6 Clinical correlation and sample size considerations

While our study focuses on the cellular and spatial mechanisms of estrogen-associated tissue remodeling, we acknowledge that clinical parameters such as POP-Q stage, patient age, and menopausal duration may influence endogenous estrogen levels and potentially modulate the formation of the HAS1+ tissue-modulatory niche. Due to the limited sample size of our discovery cohort (n=11), robust correlation analyses between the tissue-modulatory niche signature and these clinical variables are not feasible. Nonetheless, we observed that the HAS1+ fibroblast proportion was consistently elevated in all estrogen-treated patients regardless of their baseline POP-Q stage or age, suggesting this niche alteration correlates more closely with exogenous estrogen exposure than with baseline disease severity alone. Future validation studies with larger, independent cohorts are needed to assess whether baseline clinical characteristics predict responsiveness to estrogen use.

## 4 Conclusion and Perspectives

This study, through the integration of single-cell and spatial multi-omics, systematically characterizes estrogen-mediated remodeling of the vaginal wall microenvironment in postmenopausal POP patients. Our findings are consistent with a coherent mechanistic framework outlining a sequential cascade of estrogen-associated tissue modulation. The cascade begins with precise cell state reprogramming, where estrogen is associated with fibroblast differentiation away from a pro-fibrotic fate and toward the HAS1+ tissue-modulatory phenotype (19). Spatially, estrogen is associated with the localization of these reparative cells to perivascular regions, where they appear to form a structured niche with pericytes (16). Finally, estrogen is associated with enhanced coordinated intercellular communication, which may strengthen fibroblast–pericyte signaling to convert spatial proximity into functional synergy that supports tissue remodeling and repair (26, 41).

This multi-level integrated model not only offers a plausible explanatory framework for understanding the individual variation in the clinical efficacy of estrogen from cellular and spatial perspectives but, more importantly, offers an updated research perspective. Future research should focus on: (1) utilizing tools such as gene editing and organoids in cellular and animal models to further explore the regulatory roles of core regulatory factors in driving tissue-modulatory differentiation and spatial homing (51); (2) developing niche-targeted precision strategies, including small molecules that promote the reparative phenotype, biomaterials that enhance cell recruitment (49, 50), and designing optimal estrogen subtype combination therapies based on computationally predicted cell-specific pharmacological profiles (28); and (3) validating the multi-omics signature established in this study across larger cohorts (13) to construct a biomarker system capable of predicting patient responsiveness to estrogen use (9, 30).

Ultimately, this study offers an additional spatial biological insight for understanding estrogen’s action and provides a hypothesis-generating reference for future pelvic floor regenerative research. However, the computational predictions regarding differential estrogen subtype effects on specific cell types require experimental validation before any translational applications can be considered.

## 5 Methods

### 5.1 Ethics statement and sample collection

This study was approved by the Institutional Review Board of Shanxi Bethune Hospital, and written informed consent was obtained from all participants. A total of 11 postmenopausal women with pelvic organ prolapse (POP) were enrolled, including 5 untreated patients (Prolapse group) and 6 patients treated with topical promestriene (Prolapse+Estrogen group) once daily for 6 weeks. Vaginal anterior wall tissues were collected during transvaginal reconstructive surgery. During the procedure, redundant, relaxed full-thickness anterior vaginal wall tissue was excised as part of standard surgical correction. This excised tissue consists predominantly of the mucosal epithelial layer, while the stromal and muscular layers are relatively thin due to prolapse-associated stretching and tissue remodeling. All samples were immediately processed for single-cell and spatial transcriptomic analysis.

### 5.2 Statistical analysis

Continuous variables were compared using the Mann-Whitney U test, and categorical variables were compared using Fisher’s exact test. SPSS software was used, with P < 0.05 considered statistically significant.

### 5.3 Tissue dissociation and single-cell suspension preparation

Fresh tissue samples were stored in sCelLiveTM Tissue Preservation Solution (Singleron) on ice within 30 minutes after surgery. Tissues were washed three times with Hanks Balanced Salt Solution (HBSS), minced into small pieces, and digested with 3 mL sCelLiveTM Tissue Dissociation Solution (Singleron) using the Singleron PythoNTM Tissue Dissociation System at 37 °C for 15 minutes. The cell suspension was filtered through a 40-μm sterile strainer. Red blood cells were lysed using GEXSCOPE^®^ Red Blood Cell Lysis Buffer (RCLB, Singleron) at a volume ratio of 1:2 (cell pellet:RCLB) for 5–8 minutes at room temperature. The mixture was centrifuged at 300 × g at 4 °C for 5 minutes, and the pellet was resuspended in PBS. Cell viability was assessed using Trypan Blue staining and microscopic evaluation.

### 5.4 Single-cell RNA sequencing library preparation

Single-cell suspensions were adjusted to a concentration of 2 × 10^5 cells/mL in PBS. Cells were loaded onto a microwell chip using the Singleron Matrix^®^ Single Cell Processing System. Barcoding beads were collected from the chip, and reverse transcription was performed to synthesize cDNA from mRNA captured by the beads. The cDNA was amplified by PCR, fragmented, and ligated with sequencing adapters using the GEXSCOPE^®^ Single Cell RNA Library Kit (Singleron). Libraries were quantified, diluted to 4 nM, pooled, and sequenced on an Illumina NovaSeq 6000 platform with 150 bp paired-end reads.

### 5.5 Spatial transcriptomics library preparation

Formalin-fixed, paraffin-embedded (FFPE) vaginal tissue sections were cut at 5 μm thickness and mounted onto Visium HD slides (10x Genomics). Deparaffinization, decrosslinking, and antigen retrieval were performed following the manufacturer’s protocol for FFPE samples. Tissue permeabilization was optimized to release RNA, which was captured by spatially barcoded oligonucleotides on the slide. cDNA synthesis, amplification, and library construction were performed according to the Visium HD Spatial Gene Expression protocol for FFPE (10x Genomics). Libraries were quantified and sequenced on an Illumina NovaSeq 6000 platform. Visium HD spatial transcriptomics was performed on one representative sample from the control group and one representative sample from the estrogen-treated group. The Visium HD slide contains 6.5 mm × 6.5 mm capture areas, with each area consisting of 2 μm × 2 μm barcoded spots aggregated into 8 μm × 8 μm square bins for data processing. Spatial dimensions are consistent across all samples.

### 5.6 Sequencing

Both scRNA-seq and spatial transcriptomics libraries were sequenced on the Illumina NovaSeq 6000 system using 150 bp paired-end reads. Sequencing depth aimed for at least 50,000 reads per cell for scRNA-seq and 50,000 reads per spot for spatial transcriptomics.

### 5.7 Single-cell RNA-seq data processing

Raw sequencing data were processed using the CeleScope pipeline (v1.9.0, Singleron). Briefly, low-quality reads and adapter sequences were trimmed using Cutadapt (v1.17). Cell barcodes and UMIs were extracted and corrected. Clean reads were aligned to the GRCh38 reference genome (Ensembl version 92) using STAR (v2.6.1a). Gene expression counts were generated using featureCounts (v2.0.1). The output was a gene-by-cell expression matrix for downstream analysis.

### 5.8 Quality control and clustering of scRNA-seq data

The Seurat R package (v5.0) was used for quality control and clustering. Cells with fewer than 200 genes or more than the top 2% of gene counts were filtered out. Mitochondrial gene content exceeding 10% was also excluded. After filtering, 116,775 high-quality cells were retained. Gene expression was normalized using the NormalizeData function, and variable genes were identified with FindVariableFeatures (top 2000 genes). Batch effects across patient samples were corrected using Seurat v5.0 Reciprocal PCA (RPCA) integration to remove inter-individual technical variation. Principal component analysis (PCA) was performed, and the top 20 principal components were used for clustering with the FindClusters function (resolution = 0.5). Cell clusters were visualized using UMAP.

### 5.9 Cell type annotation

Cell types were annotated based on the expression of canonical marker genes from the SynEcoSys™ database (Singleron) and published references. Major cell types identified included epithelial cells, endothelial cells, fibroblasts, Pericytes, T cells, mast cells, and mononuclear phagocytes. Fibroblast subclusters were further annotated using subsetting and reclustering.

### 5.10 Differential expression and pathway enrichment analysis

Differentially expressed genes (DEGs) between groups were identified using the FindMarkers function in Seurat with the Wilcoxon rank-sum test. Genes expressed in more than 10% of cells in both groups and with an average log2 fold change > 0.25 were considered significant. Adjusted p-values were calculated using Bonferroni correction. Pathway enrichment analysis was performed using the clusterProfiler R package (v3.16.1) for Gene Ontology (GO) and Kyoto Encyclopedia of Genes and Genomes (KEGG) terms. Pathways with adjusted p-value < 0.05 were considered significantly enriched.

### 5.11 Trajectory inference analysis

Cell differentiation trajectories were inferred using CytoTRACE (v0.3.3) to estimate differentiation potential. Pseudotime analysis was performed with Monocle2 (v2.22.0) on fibroblast subpopulations. Monocle2 was selected because its DDRTree algorithm is well-suited for reconstructing bifurcating trajectories with clear branch points, which aligns with our biological question of fibroblast state transition (52, 53). Highly variable genes were used to order cells along trajectories, and branch points were visualized using UMAP.

### 5.12 Cell-cell interaction analysis

CellChat (v0.0.2) was used to infer intercellular communication networks. Ligand-receptor interactions were evaluated based on a curated database. Interaction strength was calculated and visualized using circle plots or heatmaps. Specific interactions between fibroblast subpopulations and Pericytes were highlighted.

### 5.13 Spatial transcriptomics data processing

Spatial gene expression data were processed using Space Ranger (10x Genomics) to generate count matrices. Data were analyzed in Seurat (v5.0) using the Load10X Spatial function. Spots with fewer than 10 UMI counts were filtered out. Gene expression was normalized and scaled. Dimensionality reduction was performed using PCA, and clustering was done with FindClusters. Spatial gene expression was visualized using SpatialFeaturePlot.

### 5.14 Integration of scRNA-seq and spatial transcriptomics data

Integration was performed using the Seurat integration pipeline. Anchor points between scRNA-seq and spatial data were identified with FindTransferAnchors. Cell type labels from scRNA-seq were transferred to spatial spots using TransferData. The proportion of cell types per spot was calculated and visualized with SpatialDimPlot. Spatial deconvolution was also validated using RCTD (Robust Cell Type Decomposition) in doublet mode.

### 5.15 Statistical analysis

All statistical analyses were performed in R (v4.1.0). Group comparisons were conducted using non-parametric tests (Wilcoxon test for two groups). P-values < 0.05 were considered statistically significant. Visualization was done using ggplot2, pheatmap, and Seurat plotting functions.

### 5.16 Single-cell drug susceptibility assessment

To assess drug sensitivity profiles at single-cell resolution, we performed computational analysis using the R package Beyondcell (version 1.2.1). Drug perturbation signatures from the built-in Beyondcell database were applied to our scRNA-seq dataset. Data preprocessing, including normalization and correction for the number of detected genes per cell, was conducted following the developer’s recommendations. The analysis generated a Beyondcell score for each cell-drug pair, which quantifies the predicted sensitivity.

### 5.17 Histological Analysis (H&E staining)

For histological evaluation, portions of fresh vaginal anterior wall tissues were fixed in 4% paraformaldehyde for 24 hours at room temperature, followed by dehydration, clearing in xylene, and embedding in paraffin. Tissue sections were cut at 5 μm thickness using a microtome (Leica, Germany). After deparaffinization and rehydration, sections were stained with Harris Hematoxylin (Sigma-Aldrich) for 5 minutes, rinsed, differentiated in 1% acid ethanol, and blued in Scott’s tap water. Eosin Y (Sigma-Aldrich) was applied for 2 minutes as a counterstain. Sections were then dehydrated, cleared, and mounted with neutral balsam (Sigma-Aldrich). Slides were scanned using a digital slide scanner (e.g., Nikon Eclipse Ci-L) or imaged with a bright-field microscope (e.g., Olympus BX53). H&E staining was used to verify the integrity of full-thickness vaginal wall architecture, including the epithelium, lamina propria, and muscularis layers.

### 5.18 Multiplex immunofluorescence staining and imaging

Multiplex immunofluorescence (mIF) staining was performed on formalin-fixed, paraffin-embedded (FFPE) vaginal wall sections using tyramide signal amplification (TSA) to validate spatial localization of target proteins. Sections were deparaffinized, rehydrated, and subjected to antigen retrieval. After blocking, sequential staining was carried out for three targets: HAS1, α−SMA, and DDR2. Primary antibodies were incubated overnight at 4 °C at the following dilutions: anti−HAS1 (Boster, A04784, rabbit, 1:500), anti−α−SMA (Servicebio, GB121364, mouse, 1:5000), and anti−DDR2 (Servicebio, GB112568, rabbit, 1:5000). Each staining cycle was followed by incubation with HRP−conjugated secondary antibody and fluorophore−conjugated TSA reagent. Antibody stripping was performed between cycles to avoid cross−reactivity. Nuclei were counterstained with DAPI, autofluorescence was quenched, and sections were mounted with anti−fade medium. Slides were scanned using a digital slide scanner, and high−resolution multispectral images were acquired for co−localization analysis.

## Data Availability

The datasets presented in this study can be found in online repositories. The names of the repository/repositories and accession number(s) can be found below: PRJNA1347381 (Bioproject, NCBI).

## Supplementary material

Supplementary Table 1 Major cell types identified by scRNA-seq and their canonical marker genes. The table lists the seven major cell types, their abbreviations, and well-established marker genes used for cell type annotation in the integrated single-cell and spatial atlas of the postmenopausal POP vaginal wall.

Supplementary Table 2 Cellular composition of individual samples at single-cell resolution.

Supplementary Table 3 Summary of cell counts and proportions by group.

Supplementary Table 4 Raw interaction counts between fibroblast subpopulations and pericytes.

Supplementary Figure 1 Subpopulation identification and characterization of endothelial cells. **(A)** UMAP plot of endothelial cell subclusters. Each dot represents one cell, and different colors indicate distinct EC subpopulations. **(B)** Stacked bar plot showing the proportion of each sample in EC subpopulations. **(C)** Stacked bar plot showing the proportion of control and estrogen-treated groups in EC subpopulations. **(D)** Heatmap of differentially expressed marker genes for each EC subpopulation. **(E)** Violin plots of key marker genes showing expression distribution across EC subpopulations. **(F)** Heatmap of Ro/E ratios for EC subpopulations. **(G)** Dot plot of the top 30 ligands expressed by ECs. **(H)** Dot plot of the top 30 receptors expressed by ECs.

Supplementary Figure 2 Identification of immune cell subpopulations and estrogen-induced immune microenvironment remodeling. **(A)** UMAP plot of immune cell subclusters identifying four subpopulations: plasma cells, helper T cells, effector CD8+ T cells, and exhausted CD8+ T cells. **(B)** Stacked bar plot showing the proportion of immune cell subpopulations in each sample. **(C)** Stacked bar plot showing the average proportion of immune cell subpopulations in control and estrogen-treated groups. **(D)** Heatmap of differentially expressed marker genes for each immune subpopulation. **(E)** Violin plots of representative marker genes verifying the specificity of subpopulation annotation. **(F)** Volcano plot of differentially expressed genes in immune cells. **(G)** GO enrichment analysis of upregulated genes. **(H)** GO enrichment analysis of downregulated genes. **(I)** KEGG pathway analysis of upregulated genes. **(J)** KEGG pathway analysis of downregulated genes.

Supplementary Figure 3 Differential gene expression analysis of immune cell subpopulations. **(A)** Heatmap of differentially expressed genes in plasma cells. **(B)** Heatmap of differentially expressed genes in helper T cells. **(C)** Heatmap of differentially expressed genes in CD8+ T cells. **(D)** Volcano plot of differentially expressed genes in plasma cells. **(E)** Volcano plot of differentially expressed genes in helper T cells. **(F)** Volcano plot of differentially expressed genes in CD8+ T cells.

Supplementary Figure 4 Subpopulation identification and functional analysis of mononuclear phagocytes. **(A)** UMAP plot of mononuclear phagocyte subclusters identifying four subpopulations: Neutrophils, Macrophages, ClassicalMono, and cDC2. **(B)** Stacked bar plot showing the proportion of each subpopulation across samples. **(C)** Stacked bar plot showing the average proportion of subpopulations in control and estrogen-treated groups. **(D)** Heatmap of differentially expressed marker genes for each MPs subpopulation. **(E)** Violin plots of representative marker genes verifying subpopulation annotation specificity. **(F)** Volcano plot of differentially expressed genes in MPs. **(G)** GO enrichment analysis of upregulated genes. **(H)** GO enrichment analysis of downregulated genes. **(I)** KEGG pathway analysis of upregulated genes. **(J)** KEGG pathway analysis of downregulated genes.

Supplementary Figure 5 Functional heterogeneity and signature scoring of fibroblast subpopulations. **(A)** Boxplot of myofibroblast signature scores showing differences in pro-fibrotic and contractile functions among fibroblast subpopulations. **(B)** Boxplot of extracellular matrix remodeling signature scores showing functional differences in matrix synthesis and remodeling. **(C)** Boxplot of inflammatory signature scores showing immune regulatory functions among subpopulations. **(D)** Boxplot of proliferation signature scores showing proliferative capacity differences. **(E)** Boxplot of pro-angiogenic signature scores showing pro-angiogenic functions. **(F)** Boxplot of pro-epithelial growth signature scores showing functions in epithelial repair.

Supplementary Figure 6 High-resolution heatmaps of spatially variable genes (corresponding to **Figure 4D**).

Supplementary Figure 7 Differentially expressed genes and functional enrichment analysis of pericytes. **(A)** Heatmap of differentially expressed genes in pericytes between estrogen-treated and control groups. **(B)** Volcano plot of differentially expressed genes in pericytes. **(C)** GO enrichment analysis of upregulated genes. **(D)** GO enrichment analysis of downregulated genes. **(E)** KEGG pathway analysis of upregulated genes. **(F)** KEGG pathway analysis of downregulated genes.
