# Supplementary figures and images for: Single-cell and spatial multi-omics reveal estrogen-mediated vaginal wall microenvironment remodeling and a perivascular reparative niche in postmenopausal pelvic organ prolapse
Source: Front Immunol. 2026 Jul 3;17:1794699. doi: 10.3389/fimmu.2026.1794699 (PMC13375785; doi:10.3389/fimmu.2026.1794699)

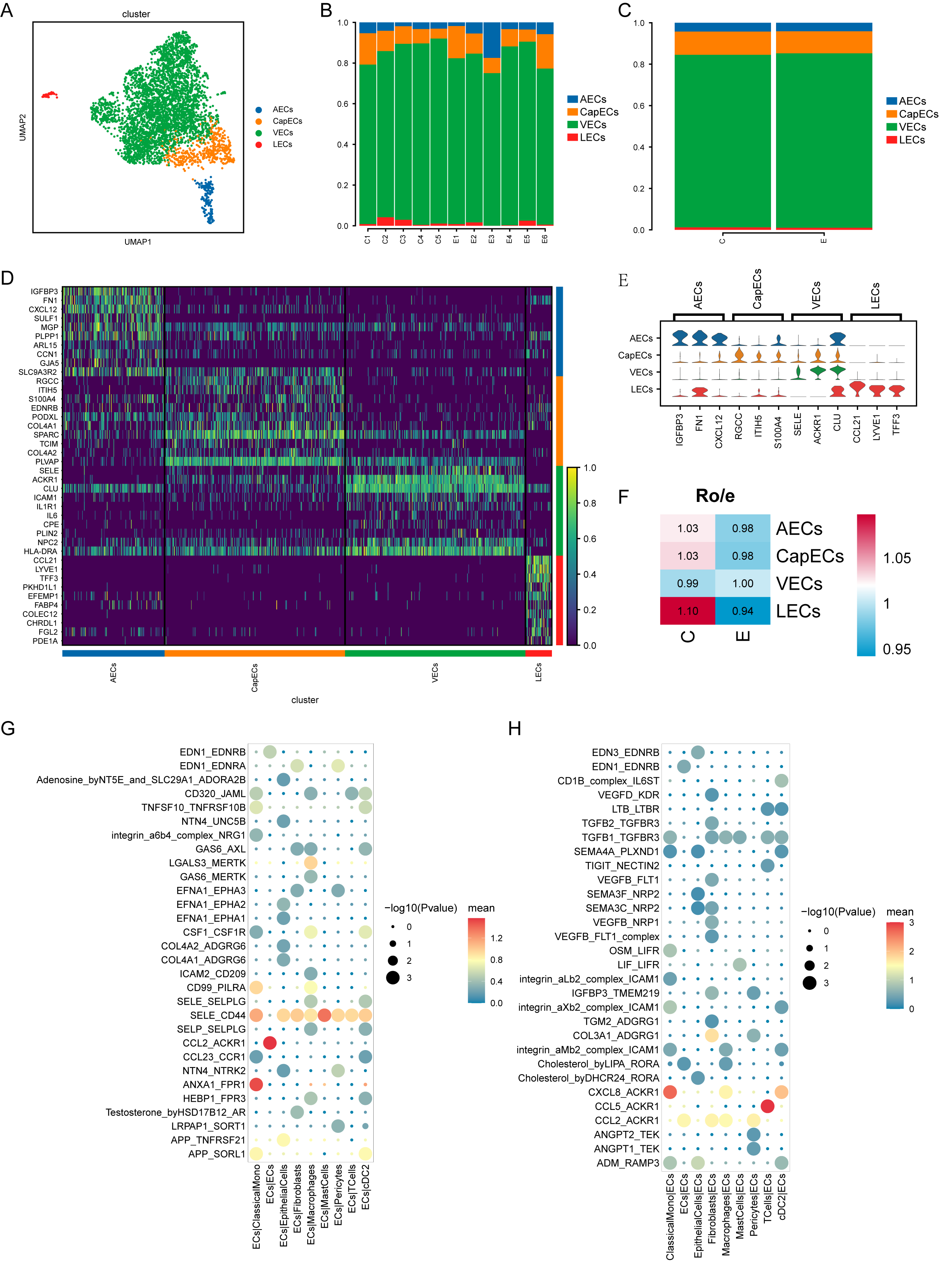

Supplement: Supplementary Table 1 — Major cell types identified by scRNA-seq and their canonical marker genes. The table lists the seven major cell types, their abbreviations, and well-established marker genes used for cell type annotation in the integrated single-cell and spatial atlas of the postmenopausal POP vaginal wall. [file Image1.tif]

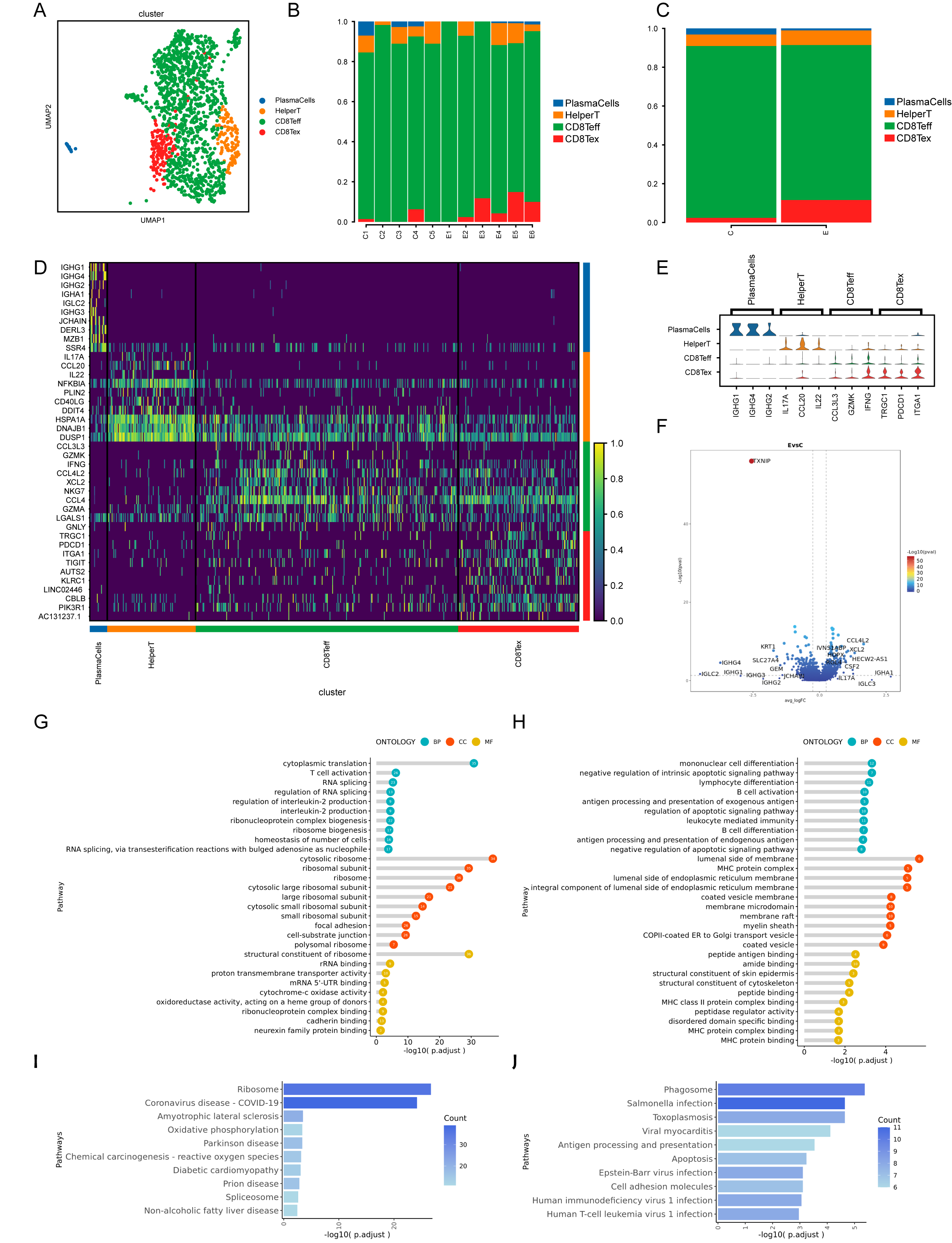

Supplement: Supplementary Table 2 — Cellular composition of individual samples at single-cell resolution. [file Image2.tif]

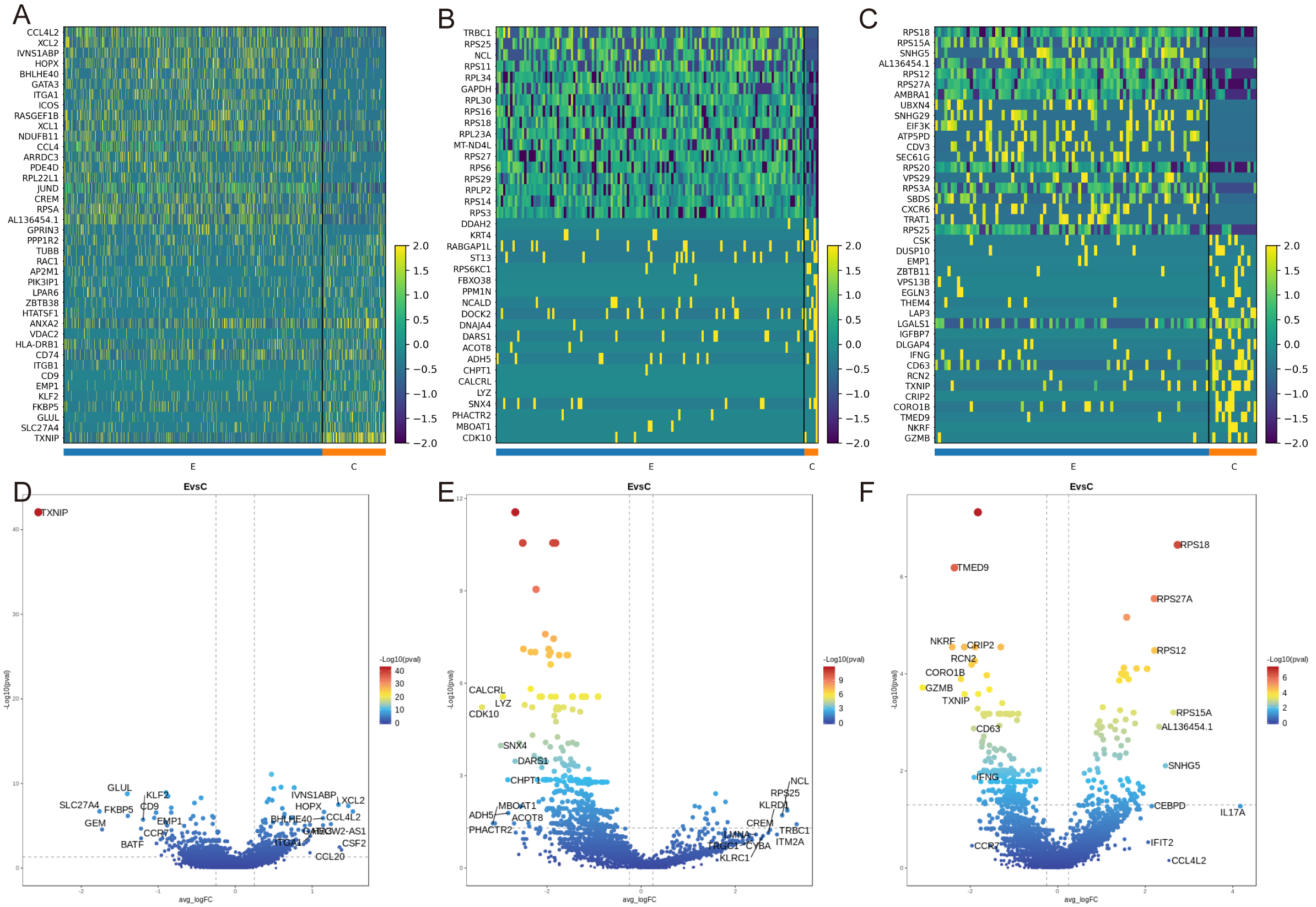

Supplement: Supplementary Table 3 — Summary of cell counts and proportions by group. [file Image3.tif]

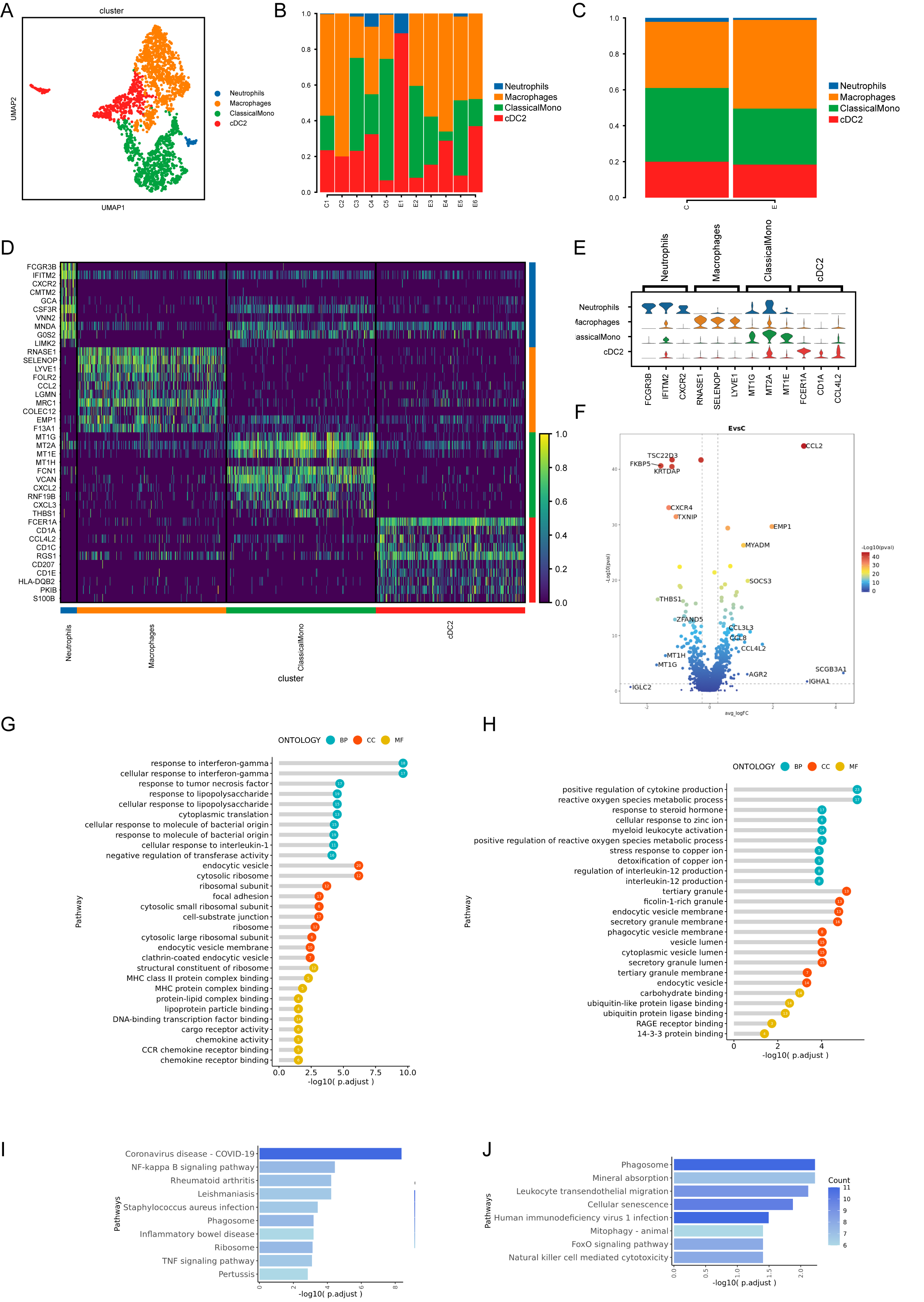

Supplement: Supplementary Table 4 — Raw interaction counts between fibroblast subpopulations and pericytes. [file Image4.tif]

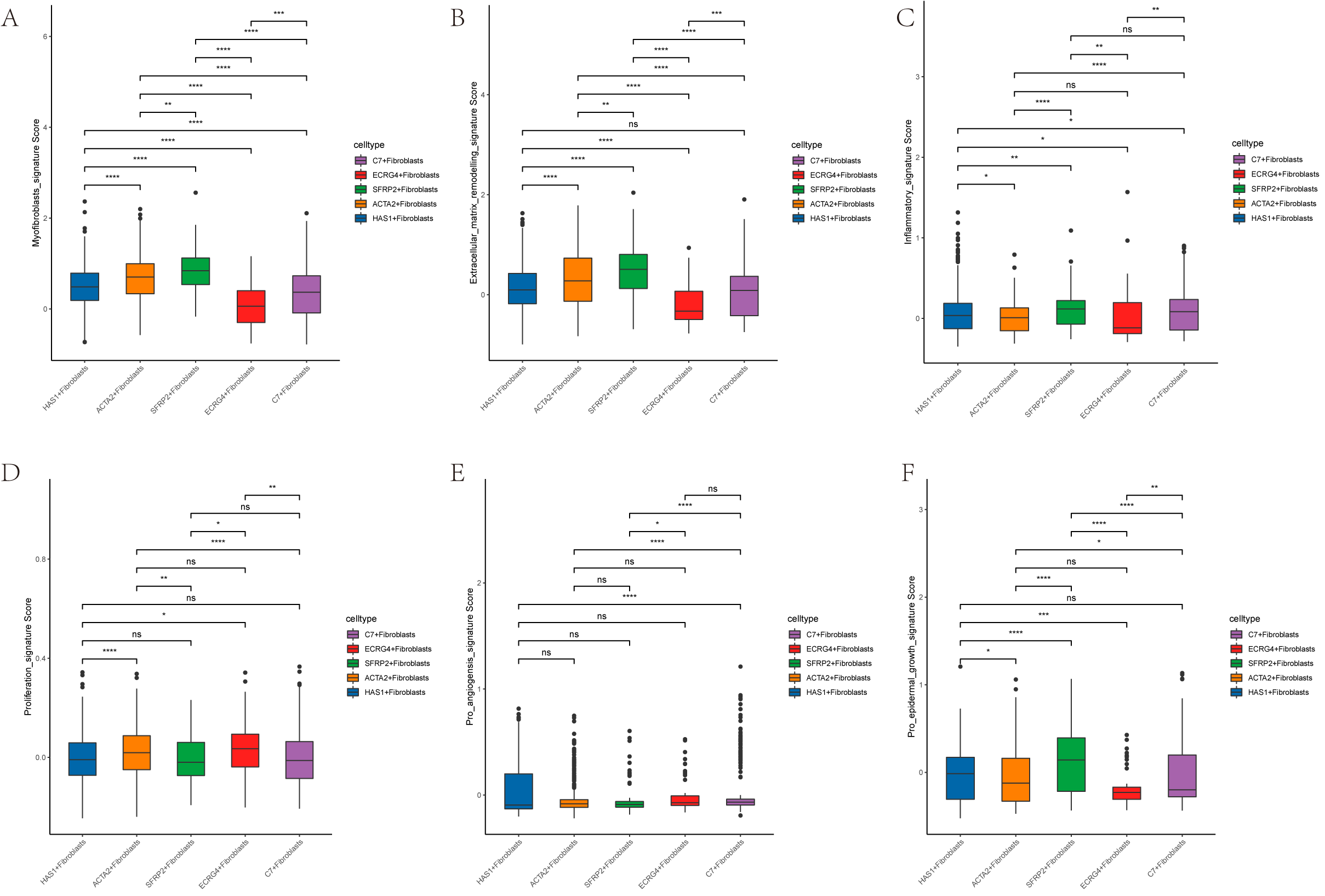

Supplement: Supplementary Figure 5 — Functional heterogeneity and signature scoring of fibroblast subpopulations. (A) Boxplot of myofibroblast signature scores showing differences in pro-fibrotic and contractile functions among fibroblast subpopulations. (B) Boxplot of extracellular matrix remodeling signature scores showing functional differences in matrix synthesis and remodeling. (C) Boxplot of inflammatory signature scores showing immune regulatory functions among subpopulations. (D) Boxplot of proliferation signature scores showing proliferative capacity differences. (E) Boxplot of pro-angiogenic signature scores showing pro-angiogenic functions. (F) Boxplot of pro-epithelial growth signature scores showing functions in epithelial repair. [file Image5.tif]

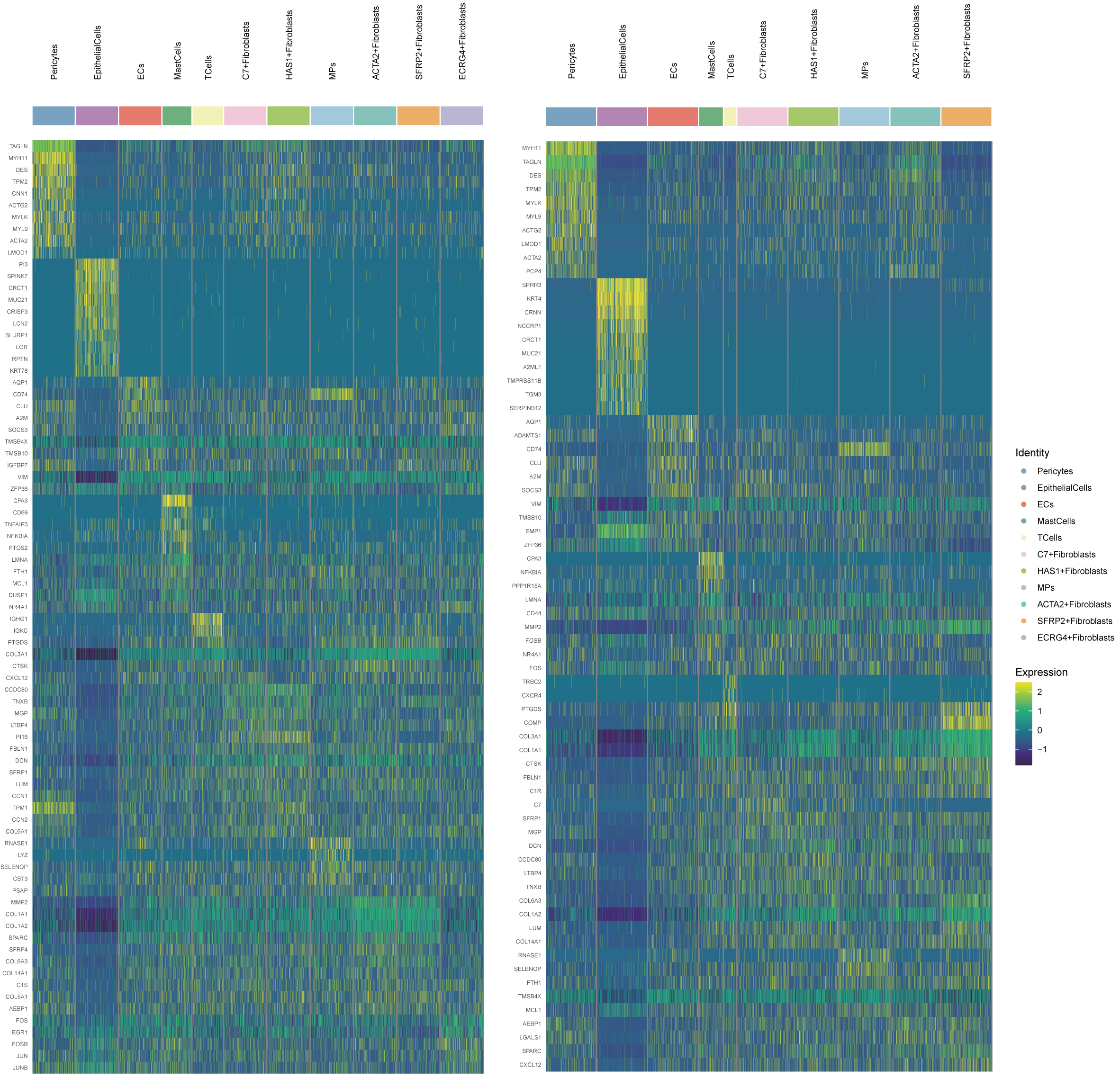

Supplement: Supplementary Figure 6 — High-resolution heatmaps of spatially variable genes (corresponding to Figure 4D). [file Image6.tif]

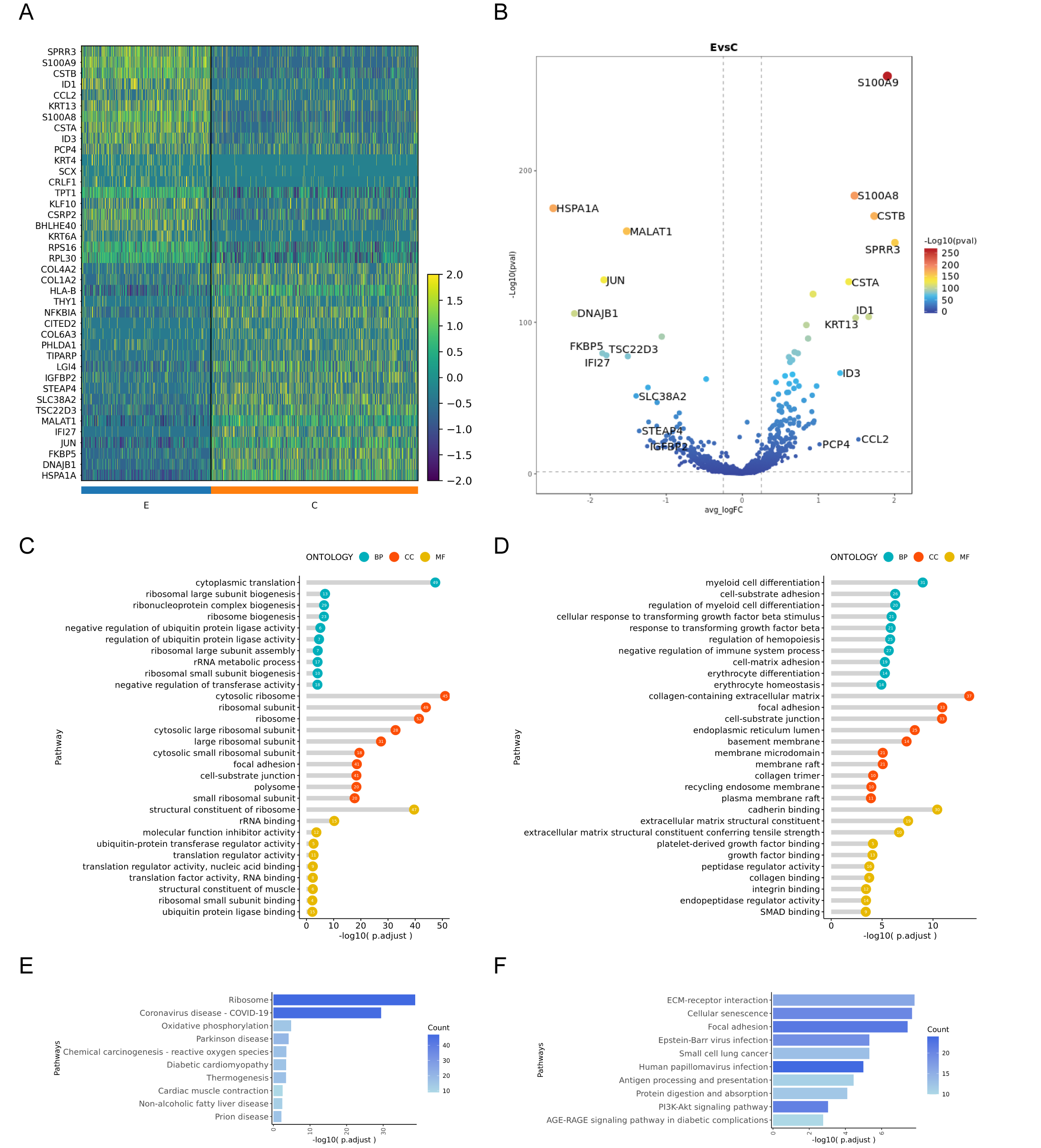

Supplement: Supplementary Figure 7 — Differentially expressed genes and functional enrichment analysis of pericytes. (A) Heatmap of differentially expressed genes in pericytes between estrogen-treated and control groups. (B) Volcano plot of differentially expressed genes in pericytes. (C) GO enrichment analysis of upregulated genes. (D) GO enrichment analysis of downregulated genes. (E) KEGG pathway analysis of upregulated genes. (F) KEGG pathway analysis of downregulated genes. [file Image7.tif]
