# Supplementary material for: Single-cell and spatial multi-omics reveal estrogen-mediated vaginal wall microenvironment remodeling and a perivascular reparative niche in postmenopausal pelvic organ prolapse
Source: Front Immunol. 2026 Jul 3;17:1794699. doi: 10.3389/fimmu.2026.1794699 (PMC13375785; doi:10.3389/fimmu.2026.1794699)
Supplement: Supplementary file 8 [file Table1.docx]

| **Supplementary Table 1. Major cell types identified by scRNA-seq and their canonical marker genes.** | | |
| --- | --- | --- |
| celltype | Abbreviation | Markers |
| Epithelial cells | EpithelialCells | EPCAM,KRT19,KRT18 |
| Endothelial cells | ECs | PECAM1,VWF,CDH5 |
| Fibroblasts | Fibroblasts | DCN,COL1A1,COL1A2 |
| Pericytes | Pericytes | ACTA2,TAGLN,RGS5 |
| T cells | TCells | CD3D,CD3E,CD3G |
| Mast cells | MastCells | TPSAB1,CPA3,TPSB2 |
| Mononuclear phagocytes | MPs | CD14,LYZ,HLA-DRA |

The table lists the seven major cell types, their abbreviations, and well-established marker genes used for cell type annotation in the integrated single-cell and spatial atlas of the postmenopausal POP vaginal wall.

| **Supplementary Table 2. Cellular composition of individual samples at single-cell resolution.** | | | | | | | | |
| --- | --- | --- | --- | --- | --- | --- | --- | --- |
| **cluster** | **EpithelialCells** | **ECs** | **Fibroblasts** | **Pericytes** | **TCells** | **MastCells** | **MPs** | **SUM** |
| C1 | 5524 (64.54%) | 784 (9.16%) | 266 (3.11%) | 1550 (18.11%) | 71 (0.83%) | 90 (1.05%) | 274 (3.20%) | 8559 |
| C2 | 12035 (97.88%) | 120 (0.98%) | 7 (0.06%) | 47 (0.38%) | 57 (0.46%) | 20 (0.16%) | 10 (0.08%) | 12296 |
| C3 | 14022 (96.38%) | 115 (0.79%) | 13 (0.09%) | 143 (0.98%) | 36 (0.25%) | 44 (0.30%) | 175 (1.20%) | 14548 |
| C4 | 5600 (84.25%) | 244 (3.67%) | 120 (1.81%) | 347 (5.22%) | 82 (1.23%) | 54 (0.81%) | 200 (3.01%) | 6647 |
| C5 | 7856 (85.49%) | 289 (3.15%) | 232 (2.52%) | 112 (1.22%) | 35 (0.38%) | 376 (4.09%) | 289 (3.15%) | 9189 |
| E1 | 8835 (93.35%) | 503 (5.31%) | 14 (0.15%) | 86 (0.91%) | 8 (0.08%) | 2 (0.02%) | 16 (0.17%) | 9464 |
| E2 | 6133 (82.33%) | 749 (10.06%) | 247 (3.32%) | 71 (0.95%) | 42 (0.56%) | 152 (2.04%) | 55 (0.74%) | 7449 |
| E3 | 6686 (96.51%) | 42 (0.61%) | 89 (1.28%) | 26 (0.38%) | 17 (0.25%) | 35 (0.51%) | 33 (0.48%) | 6928 |
| E4 | 14982 (87.10%) | 806 (4.69%) | 263 (1.53%) | 470 (2.73%) | 185 (1.08%) | 327 (1.90%) | 168 (0.98%) | 17201 |
| E5 | 8627 (76.57%) | 285 (2.53%) | 381 (3.38%) | 174 (1.54%) | 580 (5.15%) | 528 (4.69%) | 692 (6.14%) | 11267 |
| E6 | 11607 (87.75%) | 193 (1.46%) | 176 (1.33%) | 552 (4.17%) | 394 (2.98%) | 66 (0.50%) | 239 (1.81%) | 13227 |

| **Supplementary Table 3. Summary of cell counts and proportions by group.** | | |
| --- | --- | --- |
| **cluster** | **POP**  **（Control Group）** | **POP with Estrogen**  **（Estrogen-treated Group）** |
| **EpithelialCells** | 45037 (87.90%) | 56870 (86.78%) |
| **ECs** | 1552 (3.03%) | 2578 (3.93%) |
| **Fibroblasts** | 638 (1.25%) | 1170 (1.79%) |
| **Pericytes** | 2199 (4.29%) | 1379 (2.10%) |
| **TCells** | 281 (0.55%) | 1226 (1.87%) |
| **MastCells** | 584 (1.14%) | 1110 (1.69%) |
| **MPs** | 948 (1.85%) | 1203 (1.84%) |
| **SUM** | 51239 | 65536 |

| **Supplementary Table 4. Raw interaction counts between fibroblast subpopulations and pericytes.** | | | | | |
| --- | --- | --- | --- | --- | --- |
| Celltype1 | Celltype2 | count | Celltype1 | Celltype2 | count |
| ACTA2+ fibroblasts | ACTA2+ fibroblasts | 53 | ACTA2+ fibroblasts | ACTA2+ fibroblasts | 6 |
| ACTA2+ fibroblasts | C7+ fibroblasts | 38 | ACTA2+ fibroblasts | C7+ fibroblasts | 20 |
| ACTA2+ fibroblasts | ECRG4+ fibroblasts | 19 | ACTA2+ fibroblasts | ECRG4+ fibroblasts | 13 |
| ACTA2+ fibroblasts | HAS1+ fibroblasts | 111 | ACTA2+ fibroblasts | HAS1+ fibroblasts | 61 |
| ACTA2+ fibroblasts | SFRP2+ fibroblasts | 90 | ACTA2+ fibroblasts | SFRP2+ fibroblasts | 24 |
| ACTA2+ fibroblasts | Pericytes | 40 | ACTA2+ fibroblasts | Pericytes | 17 |
| C7+ fibroblasts | ACTA2+ fibroblasts | 38 | C7+ fibroblasts | ACTA2+ fibroblasts | 20 |
| C7+ fibroblasts | C7+ fibroblasts | 4 | C7+ fibroblasts | C7+ fibroblasts | 10 |
| C7+ fibroblasts | ECRG4+ fibroblasts | 8 | C7+ fibroblasts | ECRG4+ fibroblasts | 10 |
| C7+ fibroblasts | HAS1+ fibroblasts | 39 | C7+ fibroblasts | HAS1+ fibroblasts | 67 |
| C7+ fibroblasts | SFRP2+ fibroblasts | 40 | C7+ fibroblasts | SFRP2+ fibroblasts | 26 |
| C7+ fibroblasts | Pericytes | 22 | C7+ fibroblasts | Pericytes | 25 |
| ECRG4+ fibroblasts | ACTA2+ fibroblasts | 19 | ECRG4+ fibroblasts | ACTA2+ fibroblasts | 13 |
| ECRG4+ fibroblasts | C7+ fibroblasts | 8 | ECRG4+ fibroblasts | C7+ fibroblasts | 10 |
| ECRG4+ fibroblasts | ECRG4+ fibroblasts | 3 | ECRG4+ fibroblasts | ECRG4+ fibroblasts | 9 |
| ECRG4+ fibroblasts | HAS1+ fibroblasts | 24 | ECRG4+ fibroblasts | HAS1+ fibroblasts | 40 |
| ECRG4+ fibroblasts | SFRP2+ fibroblasts | 28 | ECRG4+ fibroblasts | SFRP2+ fibroblasts | 19 |
| ECRG4+ fibroblasts | Pericytes | 10 | ECRG4+ fibroblasts | Pericytes | 17 |
| HAS1+ fibroblasts | ACTA2+ fibroblasts | 111 | HAS1+ fibroblasts | ACTA2+ fibroblasts | 61 |
| HAS1+ fibroblasts | C7+ fibroblasts | 39 | HAS1+ fibroblasts | C7+ fibroblasts | 67 |
| HAS1+ fibroblasts | ECRG4+ fibroblasts | 24 | HAS1+ fibroblasts | ECRG4+ fibroblasts | 40 |
| HAS1+ fibroblasts | HAS1+ fibroblasts | 50 | HAS1+ fibroblasts | HAS1+ fibroblasts | 80 |
| HAS1+ fibroblasts | SFRP2+ fibroblasts | 90 | HAS1+ fibroblasts | SFRP2+ fibroblasts | 56 |
| HAS1+ fibroblasts | Pericytes | 42 | HAS1+ fibroblasts | Pericytes | 51 |
| SFRP2+ fibroblasts | ACTA2+ fibroblasts | 90 | SFRP2+ fibroblasts | ACTA2+ fibroblasts | 24 |
| SFRP2+ fibroblasts | C7+ fibroblasts | 40 | SFRP2+ fibroblasts | C7+ fibroblasts | 26 |
| SFRP2+ fibroblasts | ECRG4+ fibroblasts | 28 | SFRP2+ fibroblasts | ECRG4+ fibroblasts | 19 |
| SFRP2+ fibroblasts | HAS1+ fibroblasts | 90 | SFRP2+ fibroblasts | HAS1+ fibroblasts | 56 |
| SFRP2+ fibroblasts | SFRP2+ fibroblasts | 39 | SFRP2+ fibroblasts | SFRP2+ fibroblasts | 6 |
| SFRP2+ fibroblasts | Pericytes | 41 | SFRP2+ fibroblasts | Pericytes | 24 |
| Pericytes | ACTA2+ fibroblasts | 40 | Pericytes | ACTA2+ fibroblasts | 17 |
| Pericytes | C7+ fibroblasts | 22 | Pericytes | C7+ fibroblasts | 25 |
| Pericytes | ECRG4+ fibroblasts | 10 | Pericytes | ECRG4+ fibroblasts | 17 |
| Pericytes | HAS1+ fibroblasts | 42 | Pericytes | HAS1+ fibroblasts | 51 |
| Pericytes | SFRP2+ fibroblasts | 41 | Pericytes | SFRP2+ fibroblasts | 24 |
| Pericytes | Pericytes | 13 | Pericytes | Pericytes | 15 |
